# Overcoming the inhibitory microenvironment surrounding oligodendrocyte progenitor cells following experimental demyelination

**DOI:** 10.1038/s41467-021-22263-4

**Published:** 2021-03-26

**Authors:** Darpan Saraswat, Hani J. Shayya, Jessie J. Polanco, Ajai Tripathi, R. Ross Welliver, Suyog U. Pol, Richard A. Seidman, Jacqueline E. Broome, Melanie A. O’Bara, Toin H. van Kuppervelt, Joanna J. Phillips, Ranjan Dutta, Fraser J. Sim

**Affiliations:** 1 Department of Pharmacology and Toxicology, Jacob’s School of Medicine and Biomedical Sciences, University at Buffalo, Buffalo, NY USA; 2 Neuroscience Program, Jacobs School of Medicine and Biomedical Sciences, University at Buffalo, Buffalo, NY USA; 3 Department of Neuroscience, Lerner Research Institute, Cleveland, OH USA; 4 Department of Biochemistry, Radboud Institute for Molecular Life Sciences, Radboud University Medical Center, Nijmegen, The Netherlands; 5 Department of Neurological Surgery, University of California, San Francisco, CA USA

**Keywords:** Oligodendrocyte, Regeneration and repair in the nervous system

## Abstract

Chronic demyelination in the human CNS is characterized by an inhibitory microenvironment that impairs recruitment and differentiation of oligodendrocyte progenitor cells (OPCs) leading to failed remyelination and axonal atrophy. By network-based transcriptomics, we identified sulfatase 2 (Sulf2) mRNA in activated human primary OPCs. Sulf2, an extracellular endosulfatase, modulates the signaling microenvironment by editing the pattern of sulfation on heparan sulfate proteoglycans. We found that Sulf2 was increased in demyelinating lesions in multiple sclerosis and was actively secreted by human OPCs. In experimental demyelination, elevated OPC Sulf1/2 expression directly impaired progenitor recruitment and subsequent generation of oligodendrocytes thereby limiting remyelination. Sulf1/2 potentiates the inhibitory microenvironment by promoting BMP and WNT signaling in OPCs. Importantly, pharmacological sulfatase inhibition using PI-88 accelerated oligodendrocyte recruitment and remyelination by blocking OPC-expressed sulfatases. Our findings define an important inhibitory role of Sulf1/2 and highlight the potential for modulation of the heparanome in the treatment of chronic demyelinating disease.

## Abstract (web summary)

Demyelination results in impairments in oligodendrocyte progenitor cell recruitment. Here the authors identify sulfatase 1/2 as a potential modulator of myelination by modulating the microenvironment around oligodendrocyte progenitor cells.

## Introduction

Endogenous myelin repair, known as remyelination, represents a regenerative process that can restore lost neurological function and prevent disease progression in degenerative conditions such as multiple sclerosis (MS)^1^. The failure of remyelination in MS has been attributed to a failure of oligodendrocyte progenitor cell (OPC) differentiation, in part, due to the presence of quiescent OPCs in regions of chronic demyelination^2,3^. Diverse signaling pathways have been identified that prevent timely OPC differentiation in rodent models and many small molecules have been described that improve the rate of murine remyelination by targeting individual rate-limiting steps^4^. Due to the complex nature of the lesion microenvironment, multiple pathways likely converge to prevent efficient remyelination. As such, we sought to define a therapeutic approach that would modulate the lesion microenvironment en masse to exact control over several inhibitory factors simultaneously to accelerate remyelination.

The accumulation of inhibitory extracellular matrix (ECM) components such as chondroitin sulfate proteoglycans and hyaluronan are known to impair remyelination in animal models and contribute to the inhibitory environment present in chronic demyelinated lesions in MS reviewed in refs. ^5–7^. A recent analysis of MS genome-wide association studies identified numerous genes associated with the modulation of chondroitin sulfate proteoglycan (CSPG) deposition and ECM modification in general^8^. Indeed, inhibition of CSPG synthesis or chondroitinase treatment can prevent CSPG accumulation following demyelination and thereby accelerate oligodendrocyte differentiation and myelin repair^9,10^. Similarly, heparan sulfate proteoglycans (HSPGs) are deposited in both active and chronic demyelinating MS lesions^11^. HSPGs represent a class of proteoglycans that interact with several growth factors^12,13^ and, thereby, have the potential to modulate several signaling cascades operant following demyelination. The regulation of signaling by HSPGs is highly dependent on modifications to the proteoglycan glycosaminoglycan sidechains. HSPG 6-O-sulfation (6S) occurs in a tissue- and stage-specific manner^14^ and can be edited by a family of two endosulfatases. Sulf1 and Sulf2 act specifically on the trisulfated IdoA2S-GlcNS6S disaccharide residue of HSPG, with no activity on chondroitin sulfate^15,16^. Sulfatase regulation of HSPGs is associated with alterations in bioavailability and activity of several growth factors and cytokines reviewed in ref. ^17^.

We previously identified a highly correlated module of genes whose expression was conserved across species and highly enriched among activated human OPCs (hOPCs)^18^. Among these genes, *Sulf2* was among the most highly expressed and dynamically downregulated during differentiation. Herein, we describe sulfatase expression in adult OPCs following demyelination in mouse central nervous system (CNS) and in demyelinated lesions in postmortem MS brain. In mice, both Sulf1 and Sulf2 were expressed in tandem and largely restricted to oligodendrocyte lineage cells in adult CNS. Using genetic and pharmacological inhibition of sulfatases, we found that OPC-expressed sulfatases modulate their local microenvironment and facilitate HS sulfation-dependent WNT and BMP signaling. Furthermore, by conditional transgenic deletion of *Sulf1/2* in adult NG2-expressing OPCs, we demonstrate that sulfatases act to impair postmitotic oligodendrocyte formation and inhibit remyelination. Taken together, our findings define a novel therapeutic target for the acceleration of OPC recruitment and differentiation, with potential translational applications in the treatment of demyelinating disease.

## Results

### HSPG 6-O endosulfatases are highly expressed by OPCs

**Fig. 1:**
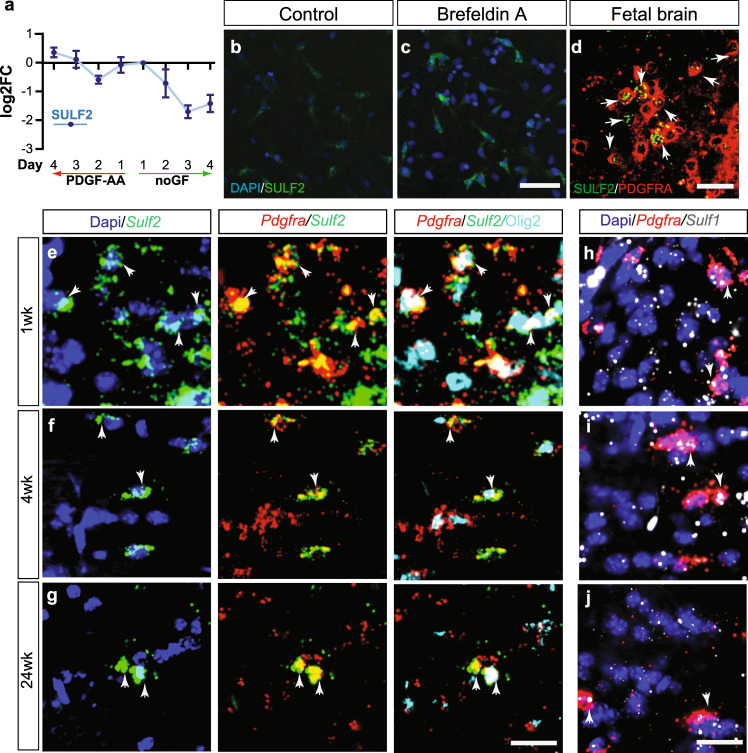
Sulfatase is expressed by mouse and human OPCs. **a** RT-PCR analysis of *SULF2* mRNA in human primary OPCs during in vitro differentiation with and without PDGF-AA removal (no GF) (mean ± SEM, *n* = 4 fetal samples). **b**, **c**, SULF2 protein (green) is expressed at low levels in hOPCs (**b**), and immunoreactivity substantially increased after treatment with the protein secretion inhibitor brefeldin A (5 µg/ml) (**c**). Confocal micrograph of *SULF2* mRNA (green)-expressing PDGFRA^+^ OPCs (red) in fetal human brain (**d**). **e**–**g** *Sulf1* and *Sulf2* expression was analyzed by confocal microscopy in mouse corpus callosum during normal development by RNAscope in situ hybridization and combined with Olig2 immunohistochemistry (IHC) at postnatal day 7 (**e**, **h**), day 28 (**f**, **i**) and at 24 weeks (**g**, **j**). DAPI (blue), *Sulf1* mRNA (gray), *Sulf2* mRNA (green), *Pdgfra* mRNA (red), and Olig2 protein (cyan). White arrows denote *Pdgfra*^+^ OPCs expressing *Sulf1* and *Sulf2*. Scale: 100 µm (**b**, **c**), 20 µm (**d**), and 20 µm (**e**–**j**).

Transcriptomic network analysis of hOPC differentiation identified a module of highly correlated and species conserved genes associated with progenitor fate^18^. Among these highly connected genes, heparan sulfate endosulfatase 2 (*SULF2*) was identified in the OPC-associated module (M5). Real-time reverse transcription-polymerase chain reaction (RT-PCR) analysis confirmed profound downregulation during hOPC in vitro differentiation (Fig. 1a). Expression of SULF2 was relatively restricted to OPCs as SULF2 messenger RNA (mRNA) expression was >14-fold higher in primary human PDGFαR^+^ OPCs than CD133^+^ neural progenitor cells^19^. Likewise, SULF2 expression was maintained in oligodendrocyte-biased PDGFαR^+^O4^+^ progenitors^20^ and adult human A2B5^+^ OPCs but not enriched in adult human astrocyte or microglial isolates^21^.

As heparan sulfate function has not been extensively studied during OPC development, we examined the expression of genes involved in HS biosynthesis, post-synthetic modifications of HS, and HSPG core proteins in mouse development (Supplementary Figure 1). We observed that several HS-associated genes were downregulated with differentiation. Downregulation of HS biosynthetic genes during OPC differentiation was consistent with previous studies that suggested reduced heparan/HS abundance during OPC differentiation^22,23^. Together, these findings suggest that the heparanome is dynamically regulated in the progenitor state and becomes more static as OPCs differentiate to mature OLs. Remarkably, both mouse *Sulf1* and *Sulf2* mRNAs were almost entirely restricted to the oligodendrocyte lineage (Supplementary Table 1). In contrast to mouse, RNA-sequencing (RNA-seq) of primary hOPCs revealed very high levels of *SULF2* expression (~100 FPKM), whereas *SULF1* mRNA was almost undetectable (<0.5 FPKM) (Supplementary Table 1). SULF2 protein expression was readily detected in hOPCs (Fig. 1b). Consistent with the active secretion of sulfatases^16,24^, blockade of the secretory pathway with brefeldin A led to cytoplasmic SULF2 protein accumulation in hOPCs (Fig. 1c).

We observed high expression of *SULF2* mRNA in vivo restricted to a subset of *PDGFRA*^+^ OPCs located in the human subventricular zone and white matter of 22-week-old fetal brain (Fig. 1d). In 1-week-old mouse brain, almost all *Pdgfra*^+^ OPCs expressed *Sulf2* mRNA (Fig. 1e and Supplementary Figure 2b), while *Sulf1* mRNA was restricted to a subset of OPCs (Fig. 1h and Supplementary Figure 2b). During early postnatal development both sulfatases were also detected in immature oligodendrocytes, but the expression in oligodendrocytes was not sustained in the adult (Fig. 1f–j). Consistent with previous reports^25^ (Allen Brain Atlas), *Sulf1* and *Sulf2* mRNAs were highly expressed within specific cortical layers (Supplementary Figure 2c–f). *Sulf2* mRNA was also expressed by a small subset of Gfap^+^ astrocytes and Iba1^+^ microglial cells (Supplementary Figure 2g–j). In the corpus callosum of aged adult mice (24 weeks), only *Pdgfra*^+^ OPCs retained high levels of *Sulf1/Sulf2* mRNA (Fig. 1g, j). Combined with our expression data in human cell isolates, these findings suggest that OPCs expressed *Sulf1*/*Sulf2* throughout developmental and into adulthood consistent with a functional role in OPC homeostasis.

### Sulfatase inhibits oligodendrocyte production following demyelination

**Fig. 2:**
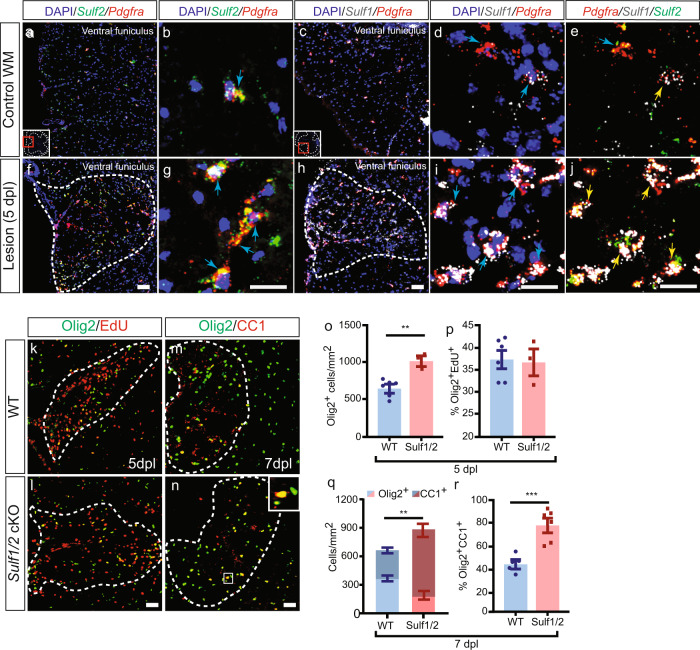
Conditional genetic ablation of OPC-expressed *Sulf1/2* accelerates oligodendrocyte recruitment and lesion colonization following demyelination. **a**–**j** Dual RNAscope in situ hybridization (ISH) of mouse spinal cord (**a**–**e**) revealed increased *Sulf1* and *Sulf2* mRNA expression following lysolecithin-induced demyelination (**f**–**j**; 5 d.p.l.). *Sulf1* (gray), *Sulf2* (green), or *Pdgfra* mRNA (red) and DAPI (blue). Yellow arrows denote *Pdgfra*^+^ OPCs that co-expresses *Sulf1/2*. Blue arrows denote *Sulf2*^+^*Pdgfra*^+^ OPCs or *Sulf1*^+^*Pdgfra*^+^ OPCs. RNAscope images were imaged by confocal microscopy, others using widefield epifluorescence. Inducible NG2creER-mediated OPC-specific ablation of *Sulf1*/*2* (*Sulf1/2* cKO) in adult mice followed by lysolecithin-induced demyelination (**k**–**r**). Analysis of OPC dynamics at 5 days post lesion (d.p.l.) (**o**–**p**) (*n* = 6 and 3 mice, for WT and *Sulf1/2* cKO respectively) and 7 d.p.l. (**q**–**r**) (*n* = 5 and 6 mice, for WT and *Sulf1/2* cKO, respectively). At 5 d.p.l., Olig2^+^ cell density was significantly increased in *Sulf1/2* cKO (**o**) (**p = 0.0016, two-sided *t* test). No difference in the proportion of proliferating EdU^+^Olig2^+^ cells compared to wild type (**p**). At 7 d.p.l., *Sulf1/2* cKO exhibited increased overall Olig2^+^ and postmitotic Olig2^+^CC1^+^ oligodendrocyte density (**m**, **n**) (cells/mm^2^ quantified in **q**, **p = 0.0027 and 0.00071, two-sided *t* test). The percentage of CC1^+^Olig2^+^ oligodendrocytes among Olig2^+^ cells was increased in *Sulf1/2* cKO animals compared to controls (**r**, ***p = 0.0009, two-sided *t* test). Mean ± SEM shown. Scale: 20 µm (**a**, **f**, **c**, **h**), 40 µm (**b**, **g**, **d**–**j**), and 10 µm (**k**–**n**).

We examined the pattern of sulfatase expression following demyelination by inducing demyelination in adult mouse spinal cord by focal injection of lysolecithin. We observed increased expression of *Sulf1* and *Sulf2* mRNA within the lesion at 5 days post lesion (5 d.p.l.) (Fig. 2a, b). At this time point, OPCs are actively recruited into the lesion. We observed that many *Pdgfra*^+^ OPCs within the lesion expressed *Sulf1* or *Sulf2* mRNAs (Fig. 2a–i), while a subset expressed both *Sulf1* and *Sulf2* (Fig. 2e, j). As in the normal brain, *Sulf2* expression was restricted to OPCs in the unlesioned spinal cord white matter. In contrast, following demyelination *Sulf2* was also expressed by a very small subset of Gfap^+^ astrocytes and Iba1^+^ microglia in the vicinity of and within the lesion, while only very few *Sulf1*^+^ astrocytes were observed (Supplementary Figure 3). Of note, Pdgfrb^+^ pericytes did not express detectable Sulf1 or Sulf2 following demyelination. These observations suggest that both sulfatases are upregulated by OPCs following demyelination and that sulfatase expression was largely restricted to OPCs and oligodendrocyte lineage cells.

To determine the functional role of OPC-expressed sulfatases following demyelination, we performed tamoxifen-induced conditional ablation of *Sulf1/Sulf2* 1 week prior to lysolecithin-induced demyelination. In order to ablate *Sulf1/2* specifically from adult OPCs, we crossed *NG2creER* mice^26^ with floxed *Sulf1/Sulf2* mice^27^. Littermate controls lacking cre expression were also injected with tamoxifen. At 5 d.p.l., we observed a significant increase in Olig2^+^ oligodendrocyte lineage cell density in *Sulf1/2* Conditional knockout (cKO) mice compared to wild type (wt) (*n* = 3–6 mice per group, *t* test *p* < 0.01) (Fig. 2k, l, o). To assess OPC proliferation, animals were terminally injected with EdU. Increased Olig2^+^ cell density was not due to increased proliferation (EdU%, *p* = 0.85) (Fig. 2p). This suggested that the ablation of sulfatase encourages a favorable microenvironment for OPC migration and recruitment.

Significantly increased density of Olig2^+^ oligodendrocyte lineage cells was maintained at 7 d.p.l. (Fig. 2m, n). At 7 d.p.l., *Sulf1/2* cKO mice exhibited a 40% increase in density of Olig2^+^ cells (*n* = 5–6 mice, *t* test *p* < 0.01) (Fig. 2q). Accompanying the increase in total oligodendrocyte lineages cells, we noted a 74% increase in the proportion of CC1^+^Olig2^+^ among Olig2^+^ cells in *Sulf1/2* cKO relative to wt controls (*p* < 0.001) (Fig. 2r). This resulted in a 2.4-fold increase in the density of Olig2^+^CC1^+^ postmitotic oligodendrocytes (*p* < 0.001). Intriguingly, the increase in oligodendrocyte differentiation at this stage resulted in the relative depletion of Olig2^+^CC1^−^-defined cells at this stage.

To more directly define progenitor recruitment following demyelination, we assessed the density of *Pdgfra* mRNA-defined OPCs and immature oligodendrocytes at each stage following demyelination and determined the rate of progenitor proliferation by co-labeling *Pdgfra*^+^ OPCs with Ki67 (Supplementary Figure 4). As shown previously^28^, in wt animals Pdgfra^+^ OPC density was greatest at 5 d.p.l. and gradually declined thereafter (Supplementary Figure 4d). Following *Sulf1/2* cKO, we observed significantly increased *Pdgfra*^+^ OPC density at both 5 and 7 d.p.l. compared to matched wt controls (Holm–Sidak multiple comparison test, *n* = 4–6 mice, *p* < 0.01) (Supplementary Figure 4d). By 14 d.p.l., the *Sulf1/*2-dependent increase in OPC density was no longer present (*n* = 3–4 per group, Holm–Sidak *p* = 0.64). The proportion of dividing Ki67^+^ OPCs likewise decreased over the same time period. Consistent with the EdU analysis above (Fig. 2P), *Sulf1/2* cKO did not influence the proportion of Ki67^+^Pdgfra^+^ OPCs at any stage following demyelination, suggesting that proliferation of OPCs was not affected by *Sulf1/2* ablation (Ki67%, Holm–Sidak *p* = 0.99) (Supplementary Figure 4e). Next, to determine, whether differential cell death contributed to the differences observed in oligodendrocyte lineage recruitment, we analyzed apoptosis via cleaved caspase-3 within the Olig2^+^ cell population (Supplementary Figure 4f–h). The differences in the proportion of apoptotic OPCs between *Sulf1/2* cKO and wt mice did not reach significance (*n* = 3–4, Holm–Sidak *p* = 0.28) (Supplementary Figure 4h) and, as such, likely do not account for the large differences in Olig2^+^ cell density observed (Fig. 2o, q). Thus, sulfatases *Sulf1/2* act to impair the recruitment and early differentiation of OPCs following demyelination.

### Individual sulfatase deletion is sufficient to improve oligodendrocyte generation following demyelination

**Fig. 3:**
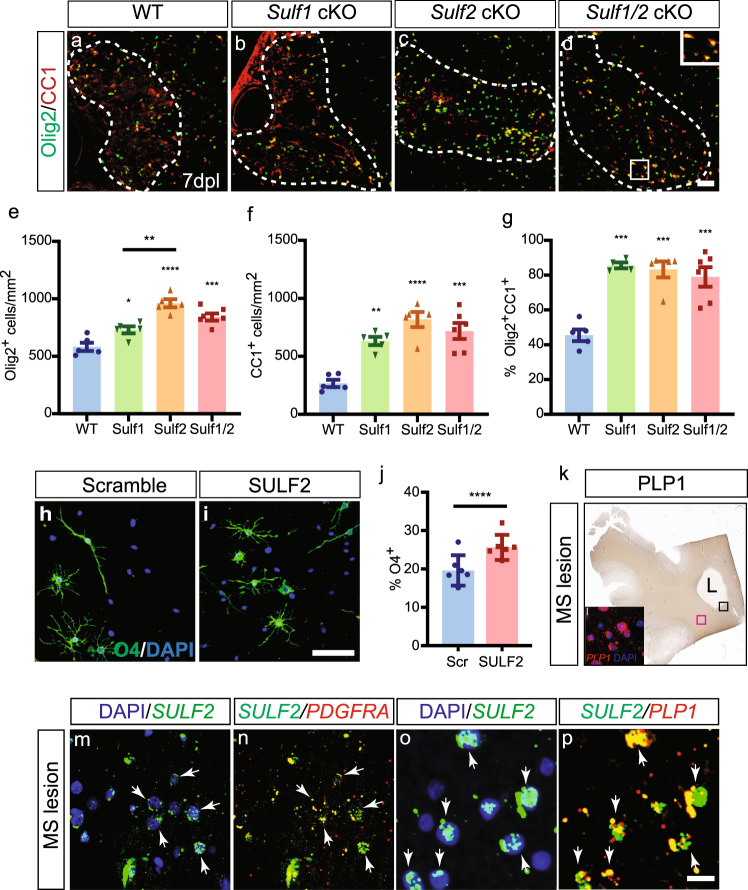
Sulf2 deletion results in improved oligodendrocyte recruitment and differentiation and is upregulated in chronic active MS lesions. Tamoxifen-dependent OPC-specific ablation of either *Sulf1*, *Sulf2* alone, or both *Sulf1/2* in NG2^+^ OPCs was initiated prior to demyelination. Control animals (lacking cre) were treated in an identical manner. Widefield immunofluorescence for Olig2 (green) and CC1 (red) at 7 d.p.l. (**a**–**d**). The density (cells/mm^2^) of Olig2^+^ oligodendrocytes lineage cells (**e**), Olig2^+^CC1^+^ postmitotic oligodendrocytes (**f**) and percentage of CC1^+^ oligodendrocytes (**g**) was quantified (*n* = 5 for each group except *n* = 6 for *Sulf1/2* cKO). Mean ± SEM is shown. Two-way ANOVA for Sulf1 and Sulf2 genotypes. Holm–Sidak post-test vs. wild-type control *, **, ***, and *** indicates *p* < 0.05, 0.01, 0.001, and 0.0001, respectively. **h**–**j** Human primary OPCs infected with lentiviral SULF2 knockdown (KD) or scrambled control cultured in mitogen-free conditions for 4 days. Oligodendrocyte differentiation assessed by O4 immunocytochemistry (green) and DAPI (blue) (**h**, **i**). **j** SULF2 KD accelerated O4^+^ oligodendrocyte differentiation (****p < 0.0001 paired *t* test, *n* = 6 fetal human samples). **k** PLP1 immunohistochemistry of chronic active human MS lesion and surrounding normal white matter (NAWM). **l** RNAscope for *PLP1* mRNA in NAWM (pink box in **k**). **m**–**p** Confocal-based RNAscope in situ hybridization at lesion border (black box in **k**) for *SULF2* (green), *PDGFRA* (red, **n**), and *PLP1* (red, **p**) mRNA. Arrow represents colocalization of *SULF2* with *PDGFRA* and *PLP1* mRNAs. Scale: 20 µm (**a**–**d**, **m**–**p**), and 50 µm (**i**, **j**).

In order to define whether the effects of *Sulf1/2* cKO were mediated specifically via deletion of either *Sulf1* or *Sulf2*, we assessed the effect of individual *Sulf1/2* cKO on OPC recruitment and differentiation following demyelination at 7 d.p.l. (*n* = 5–6 mice per genotype) (Fig. 3a–g). Interestingly, individual *Sulf2* deletion resulted in a significant increase in the density of Olig2^+^ oligodendrocyte lineage cells that resembled that of *Sulf1/2* double cKOs (Fig. 3e). In contrast, *Sulf1* deletion resulted in only a relatively mild increase to Olig2^+^ density relative to control (Fig. 3e). Indeed, two-way analysis of variance (ANOVA) treating *Sulf1/2* as dependent variables indicated a main effect of *Sulf2* but not *Sulf1* genotype on Olig2^+^ recruitment (*Sulf2* *p* < 0.0001, *Sulf2* *p* = 0.67). Consistent with a shared mechanism, a significant interaction between *Sulf1* and *Sulf2* was found (*F* (1, 17) = 15.8, *p* = 0.001) (Fig. 3e). In contrast, individual deletion of either *Sulf1* or *Sulf2* resulted in a highly significant increase in CC1^+^Olig2^+^ oligodendrocyte density relative to wt (two-way ANOVA, main effects *p* < 0.05). A highly significant interaction of *Sulf1* and *Sulf2* genotype effect on Olig2^+^CC1^+^ cell density was also consistent with a common function of either gene (*F* (1, 17) = 17.4, interaction *p* = 0.0006) (Fig. 3f). Likewise, as an estimate of differentiation, the proportion of CC1^+^Olig2^+^ cells among Olig2^+^ oligodendrocyte lineage cells was increased following ablation of either *Sulf1* or *Sulf2*, and comparable with *Sulf1/2* double cKO mice (*F* (1, 16) > 10, *p* < 0.01 for main effects and interaction) (Fig. 3g).

Together, these data suggest that *Sulf1* and *Sulf2* both contribute to delayed OPC recruitment and oligodendrocyte differentiation following demyelination. As deletion of either sulfatase resulted in substantial alterations in the density of oligodendrocytes and oligodendrocyte lineage cells, it is clear that the loss of either *Sulf1* or *Sulf2* was not fully compensated by the remaining sulfatase gene.

### *SULF2* inhibits hOPC differentiation and migration and is enriched in MS lesions

Having established that *Sulf2* can impair OPC recruitment and differentiation in mice following demyelination, we next studied the functional role of sulfatase expression in purified hOPCs. RNA-seq and quantitative PCR revealed that SULF2 was the principal sulfatase expressed by hOPCs (Supplementary Table 1 and Supplementary Figure 5a). We examined the function of SULF2 in hOPCs following infection with lentivirus expressing SULF2 short hairpin RNA (shRNA)^29^, which significantly reduced *SULF2* mRNA expression by 72.9 ± 4.7% (Supplementary Figure 5b) (*p* < 0.0001, *n* = 8 individual fetal samples). Importantly, *SULF1* mRNA expression was not increased in compensation following SULF2 knockdown (KD) (Supplementary Figure 5c) (*p* = 0.90) and remained at the lower detection limit (*C*_t_ > 30). Importantly, SULF2 KD significantly accelerated human O4^+^ oligodendroglial differentiation by 30% compared to scrambled controls (Fig. 3I, j) (*n* = 6 samples, *p* < 0.0001). Next, to assess whether SULF2 KD modulates hOPC migration and thereby may directly regulate OPC recruitment following demyelination, we assessed migration in a transwell-based assay following treatment with either WNT3 or CXCL1 (Supplementary Figure 6). SULF2 KD rescued the negative effects of either CXCL1 or WNT3A treatment on OPC migration (*n* = 5 samples, *p* < 0.05). Lastly, to determine if hOPCs actively secreted SULF2, we performed slot-blot analysis of conditioned media as well as cell lysates (Supplementary Figure 5d). Consistent with *SULF2* mRNA downregulation upon differentiation (Fig. 1a), both cellular and secreted SULF2 protein expression decreased during hOPC differentiation.

As SULF2 acted to prevent efficient hOPC differentiation in vitro, we next asked whether *SULF2* was expressed by OPCs in adult human brain and in chronic active demyelinated lesions from secondary progressive MS patients (*n* = 2). *SULF2* mRNA was expressed by *PDGFRA*^+^ OPCs in normal-appearing white matter and substantially increased around the demyelinated lesion border (Fig. 3l–p). We confirmed that these *SULF2* mRNA transcripts were restricted to OPC and oligodendrocyte cells by their colocalization with *PDGFRA* (Fig. 3m, n) and *PLP1* transcripts, respectively (Fig. 3o, p). Almost all *PDGFRA*^+^ and *PLP1*^+^ cells observed at the lesion border expressed *SULF2* mRNA. Together, these data suggest that SULF2 is upregulated in a similar manner following demyelination in the human brain and in vitro act in a similar manner to prevent efficient OPC differentiation.

### Conditional sulfatase ablation accelerates remyelination following demyelination

**Fig. 4:**
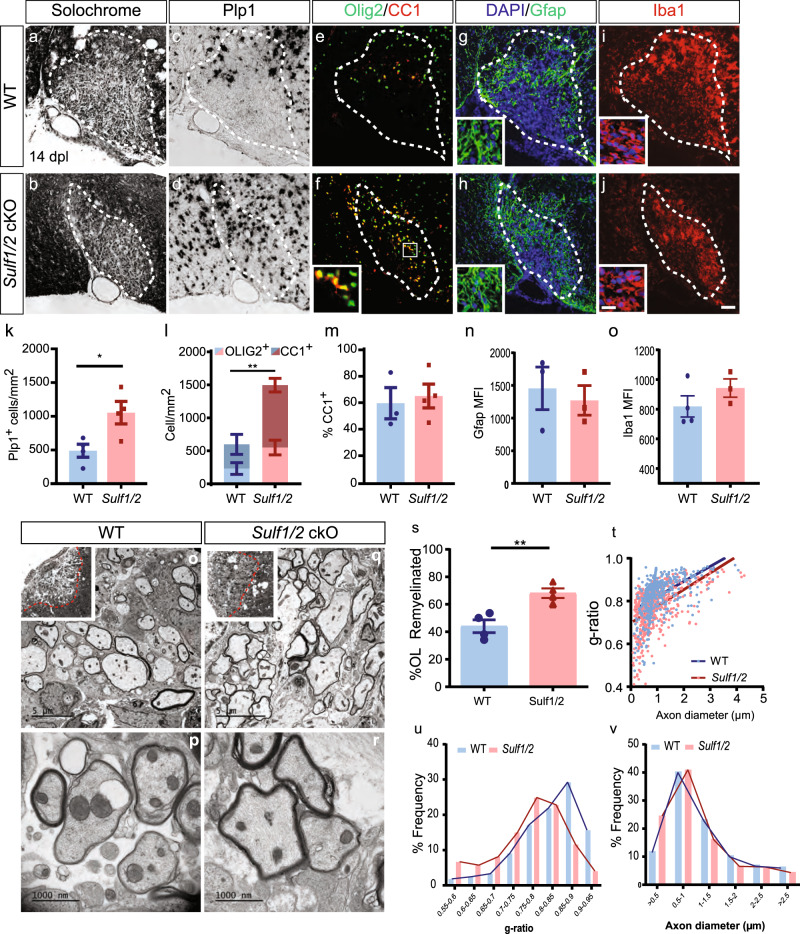
Conditional ablation of *Sulf1/2* accelerates remyelination. **a**, **b** Solochrome cyanine-stained lesions at 14 days post lesion (d.p.l.). Oligodendrocyte differentiation was assessed by *Plp1* ISH (**c**, **d**) and Olig2^+^CC1^+^ immunofluorescence (Olig2, green; CC1, red) (**e**, **f**). Astrogliosis was assessed by Gfap (green) and image insert shows a higher magnification image to show morphology (**g**, **h**), and the microglial responses were assessed by Iba1 (red) and image insert at higher magnification showing morphology (**i**, **j**). Quantification of *Plp1*^+^ oligodendrocyte cell density (**k**) (*indicates two-sided *t* test *p* = 0.026; *n* = 4 mice per group), Olig2^+^ and CC1^+^ density (**l**) (**indicates total Olig2^+^ density two-sided *t* test *p* = 0.0077; *n* = 4 mice per group), and percentage of CC1^+^ cells within the Olig2 population (**m**) (*n* = 3 and 4 mice, for WT and *Sulf1/2* cKO, respectively). **n** Mean fluorescence intensity (MFI) of Gfrap (*n* = 3 mice per group) and Iba1 cells in lesion (*n* = 4 and 3 mice, for WT and *Sulf1/2* cKO respectively). **o**–**v** Analysis of remyelination by electron microscopy at 14 d.p.l. **o**, **q** Inserts show lesion location within ventrolateral white matter. **s** Proportion of remyelinated axons (**indicates two-sided *t* test *p* = 0.0061) and **t** relationship between axon diameter and *g*-ratio (linear regression is shown). Frequency distribution of axonal *g*-ratio (**u**) and axon diameter (**v**) in lesion (*n* = 4 mice/group, ≥400 axons). Mean ± SEM shown. Scale: 20 μm (**a**–**j**) and 5 μm (**o**, **q**) and 1000 nm (**p**, **r**).

To address whether Sulf1/2 modulation of HSPG sulfation could influence oligodendrocyte density and remyelination following lysolecithin-induced demyelination, additional cohorts of *Sulf1/2* cKO and control mice were sacrificed at 14 d.p.l. As observed at 5 and 7 d.p.l., demyelinated lesions were similar in size in *Sulf1/2* cKO and control mice (*n* = 4, *t* test *p* = 0.81) (Fig. 4a, b). Sulfatase deletion resulted in a 2-fold increase in the density of *Plp1*^+^ oligodendrocytes and 2.6-fold increase in Olig2^+^CC1^+^ oligodendrocyte density (*n* = 4 per group, *p* < 0.05) (Fig. 4c, d, k, l) as seen at 7 d.p.l. Likewise, increased mature oligodendrocyte density in *Sulf1/2* cKO animals was accompanied by the increased total density of Olig2^+^ oligodendrocyte lineage cells relative to wt controls (*p* < 0.01) (Fig. 4l). The density of Olig2^+^CC1^−^ defined cells was equivalent between groups at 14 d.p.l. (Fig. 4l) as was the density of Pdgfra^+^ OPCs (Supplementary Figure 4d). The percentage of Olig2^+^CC1^+^ oligodendrocytes within the Olig2^+^ population was no longer enhanced at 14 d.p.l. (*p* = 0.72) (Fig. 4m). Taken together, *Sulf1/2* cKO resulted in accelerated OPC recruitment and differentiation of new oligodendrocytes leading to the increased density of both mature oligodendrocytes and OPCs following demyelination in the CNS.

As OPC secreted sulfatases may regulate local signaling in other cell types, we examined the effect of OPC-specific deletion of *Sulf1/2* on astrocyte and microglial activation following demyelination. We did not observe differences in the gross pattern or intensity of astrocytic Gfap immunoreactivity (*p* = 0.66) (Fig. 4g, h). Likewise, the overall distribution and intensity Iba1^+^ microglia/macrophages staining was not significantly altered by *Sulf1/2* cKO (*p* = 0.26) (Fig. 4i, j, n). Thus, while paracrine effects of OPC-expressed *Sulf1/2* cannot be excluded, there were no overt effects on the gliotic response following demyelination.

We next investigated whether decreased sulfatase activity in OPCs might accelerate remyelination (Fig. 4o–v). At 14 d.p.l., *Sulf1/2* cKO significantly increased the proportion of oligodendrocyte remyelinated axons relative to wt controls (*n* = 4 mice per group, *t* test *p* = 0.0061) (Fig. 4s). While a small proportion of axons were remyelinated by Schwann cells, we did not observe any qualitative differences in the proportion of Schwann cell myelinated axons following *Sulf1/2* cKO. Consistent with an acceleration of oligodendrocyte differentiation, myelin thickness was significantly increased in remyelinated axons following *Sulf1/2* deletion. The myelin *g*-ratio, which represents the ratio of axon to axon plus myelin diameter, was significantly decreased in *Sulf1/2* cKO (*g*-ratio calculated from *n* = 4 mice, *t* test *p* < 0.05). Linear regression analysis of individual *g*-ratio vs. axon diameter also demonstrated a significant effect of *Sulf1/2* cKO (Fig. 4t). Likewise, the distribution of *g*-ratios demonstrated a significant reduction in the frequency of very thinly myelinated axons consistent with improved remyelination initiation following *Sulf1/2* cKO (two-way ANOVA, Sidak post hoc, *g*-ratio 0.85–0.9 *p* = 0.0016 and 0.9–0.95 *p* = 0.019) (Fig. 4u). *Sulf1/2* cKO did not influence the distribution of axonal diameter within the lesion suggesting that deletion of *Sulf1/2* did not induce axonal swelling (Fig. 4v). These results indicate that OPC-expressed sulfatases act as negative regulators for remyelination in a cell-autonomous manner.

### WNT/BMP signaling inhibits oligodendrocyte differentiation following demyelination in a sulfatase-dependent manner

**Fig. 5:**
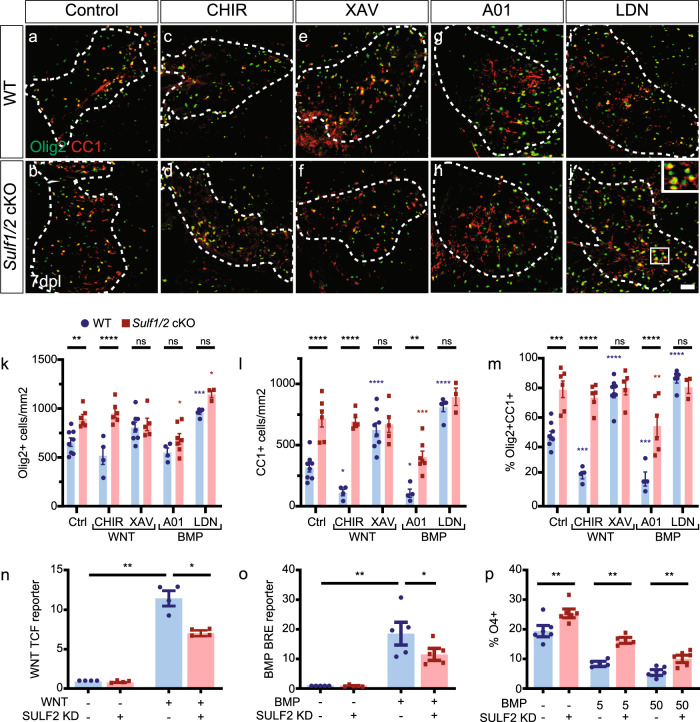
Sulfatase promotes inhibitory WNT and BMP signaling on oligodendrocyte differentiation following demyelination. Pharmacological manipulation of WNT and BMP signaling was performed in the context of Sulf*1/2* cKO. WNT pathway activator (CHIR-99021, 3 µM), WNT antagonist (XAV939, 100 nM), BMP pathway activator (A01, 100 nM), or receptor antagonist (LDN-193189, 100 nM) were co-injected with lysolecithin. **a**–**j** Oligodendrocyte lineage cell density was assessed by Olig2 (green) and CC1 (red) widefield immunofluorescence. Density of Olig2^+^ oligodendrocyte lineage cells (**k**), Olig2^+^CC1^+^ postmitotic oligodendrocyte (**l**), and percentage of CC1^+^ oligodendrocytes (**m**) quantified (**k**–**m**, *n* = 8, 5, 4, 6, 8, 5, 4, 7, 5, and 3 mice from left to right, for ctrl, CHIR, XAV, A01, and LDN treatment groups, in WT and *Sulf1/2* cKO mice respectively). Two-way ANOVA was performed (see Source data for full details). Holm–Sidak multiple comparisons tests vs. ctrl wild-type (blue *) or ctrl Sulf1/2 cKO (red *), or as indicated. *, **, ***, and **** indicated *p* < 0.05, <0.01, <0.001, and <0.0001 respectively. **n** hOPCs were co-infected with lentiviral SULF2 shRNAi or scrambled control and lentiviral TCF/LEF reporter virus and exposed to WNT3A (50 ng/mL) or vehicle control. Two-way RM ANOVA followed by Holm–Sidak post-test (*n* = 4 fetal human samples, mean ± SEM normalized to control). **o** hOPCs infected with lentiviral BMP reporter and SULF2 KD virus. SULF2 KD reduced BMP activity in hOPCs following BMP7 treatment (50 ng/mL) (*n* = 5, *p* < 0.05 two-way RM ANOVA Holm–Sidak post-test). Two-way ANOVA revealed significant main effects for ligand treatment, SULF2 KD, and the interaction for both WNT and BMP. **p** quantification of O4^+^ oligodendrocyte differentiation following SULF2 KD and BMP7 treatment (5–50 ng/mL BMP7) (*n* = 5 at 5 ng/mL BMP7 and *n* = 6 at 0 and 50 ng/mL BMP7 individual fetal human samples). SULF2 knockdown attenuated the effects of WNT/BMP signaling and significantly increased differentiation of hOPCs in the presence of BMP (mixed-effects model; Sidak’s post-test, *p* < 0.05). * and ** indicate *p* < 0.05 and < 0.01, respectively. Mean ± SEM is shown. Scale: 20 μm (**a**–**j**). n.s. Not significant.

Pathological activation of both BMP and WNT signaling prevents efficient remyelination by inhibiting OPC differentiation^30–32^. Importantly, antagonism of either WNT or BMP signaling can accelerate OPC differentiation and remyelination^33,34^. As sulfatase ablation has been shown to impair both BMP^35^ and WNT signaling^13^, we hypothesized that *Sulf1/2* cKO increased oligodendrocyte density due to modulation of WNT and BMP signaling in OPCs. To this end, we used pharmacological approaches to specifically activate or inactivate either WNT or BMP signaling in the context of *Sulf1/2* deletion at 7 d.p.l. (Fig. 5a–m). Consistent with previous studies and data presented above, two-way ANOVA revealed significant effects of drug treatment and *Sulf1/2* genotype on all parameters as well as a highly significant interaction between them (*n* = 3–8 mice per group).

As previously shown with genetic activation of WNT signaling^32^, pharmacological WNT activation via CHIR-99021 (3 µM) in wt animals resulted in significantly impaired differentiation with reduced density of CC1^+^Olig2^+^ oligodendrocytes (*n* = 4 and 8, respectively, Holm–Sidak multiple comparison test *p* < 0.05) (Fig. 5l) and reduced proportion of CC1^+^Olig2^+^ oligodendrocytes among total Olig2^+^ cells (Holm–Sidak *p* < 0.001) (Fig. 5m) following demyelination. Likewise, tankyrase inhibitor XAV939 (0.1 µM), which blocks WNT signaling, significantly promoted oligodendrocyte differentiation in wt mice measured as CC1^+^Olig2^+^ density and CC1% at 7 d.p.l. (Hold–Sidak *p* < 0.0001), as previously shown^33^. As described above, *Sulf1/2* cKO significantly increased both metrics of differentiation. In the context of *Sulf1/2* cKO, WNT activation (CHIR-99021) was unable to block oligodendrocyte differentiation (Olig2^+^CC1^+^ density *p* = 0.78, CC1% *p* = 0.76) (Fig. 5l, m). Likewise, additional WNT antagonism by XAV939 did not further potentiate the increase in oligodendrocyte differentiation afforded by *Sulf1/2* cKO (Olig2^+^CC1^+^ density *p* = 0.78, CC1% *p* = 0.97). Thus, *Sulf1/2* deletion in OPCs promoted oligodendrocyte differentiation in the presence of WNT activation but was not able to act additively with WNT antagonist treatment to further improve oligodendrocyte generation. These results are therefore consistent with the role of the *Sulf1/2* in the regulation of WNT signaling but also highlight the potential that other signaling pathways are modulated to promote oligodendrocyte generation following demyelination regardless of WNT activation.

Next, we used A01 (100 nM) to inhibit E3 ligase Smurf1 and thereby activate intracellular BMP signaling^36^ and BMP type I receptor antagonist LDN-193189 (100 nM) to antagonize BMP signaling (Fig. 5a–m). In wt animals, pharmacological BMP activation (A01) significantly decreased Olig2^+^CC1^+^ oligodendrocyte density and CC1^+^ percentage (Holm–Sidak *p* < 0.05 and *p* < 0.001) (Fig. 5l, m). In contrast, BMP receptor antagonist (LDN) increased all three parameters, total Olig2^+^ density (Holm–Sidak *p* = 0.0004) (Fig. 5k), CC1^+^ density (*p* < 0.0001) (Fig. 5l) and %CC1 (*p* < 0.0001) (Fig. 5m). In *Sulf1/2* cKO mice, intracellular BMP activation via A01 treatment reduced oligodendrocyte differentiation (Olig2^+^CC1^+^ density and CC1%) compared to *Sulf1/2* cKO alone (Fig. 5l, m). However, this level of differentiation was significantly enriched compared to wt, indicating that *Sulf1/2* cKO effect was still effective in the context of BMP excessive activation. In contrast, *Sulf1/2* cKO did not further enhance oligodendrocyte differentiation in animals treated with BMP receptor antagonist (LDN) (Hold–Sidak, Olig2^+^CC1^+^ density *p* = 0.97, CC1% *p* = 0.14) (Fig. 5l, m). The interaction between BMP receptor antagonist treatment and sulfatase suggests that sulfatase acts via BMP signaling.

As modulation of these pathways could occur at multiple levels, we next asked whether sulfatase modulated WNT and BMP signaling directly in hOPCs. To this end, scrambled control or SULF2 KD hOPCs were transduced with viral reporters for WNT and BMP signaling. WNT-dependent TCF reporter luciferase was >11-fold induced following WNT3a treatment (*n* = 4 fetal samples) (Fig. 5n). Strikingly, SULF2 KD significantly attenuated WNT induction by >35%. Likewise, BMP response element (BRE)-dependent luciferase was increased >18-fold by BMP7 treatment and this BMP-induced BRE luciferase activity was significantly inhibited by SULF2 KD (Fig. 5o). Two-way ANOVA revealed a significant interaction effect between SULF2 KD and both WNT and BMP signaling (*p* < 0.05) and was consistent with a direct effect of sulfatase on these signaling pathways. To further test whether SULF2 KD could directly influence the effects of BMP signaling on hOPC fate, we treated SULF2 KD and scrambled infected hOPCs with BMP7 and assessed the effects on O4^+^ oligodendrocyte differentiation (*n* = 5–6 fetal samples) (Supplementary Figure 7). As shown previously^37^, BMP7 treatment inhibited O4^+^ differentiation from hOPCs (two-way ANOVA, *p* < 0.0001) (Fig. 5p). Importantly, SULF2 KD significantly rescued the effects of BMP7 (5 ng/mL, Hold–Sidak test *p* = 0.0029; 50 ng/mL, *p* = 0.017). As such, sulfatase expressed by hOPCs directly potentiates both WNT and BMP signaling and is consistent with sulfatases exerting cell-autonomous role in OPCs.

### PI-88 modulates OPCs sulfation and regulates BMP and WNT signaling in hOPCs

**Fig. 6:**
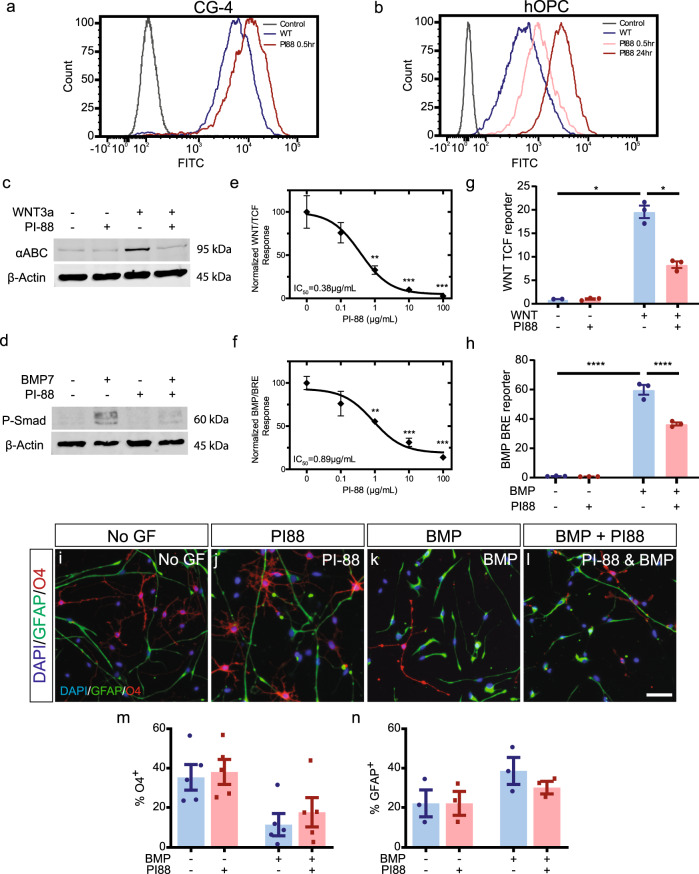
PI-88 inhibits sulfatases leading to increased HSPG sulfation, and inhibition of WNT and BMP signaling in OPCs. **a**, **b** Flow cytometry of HS sulfation using RB4CD12 antibody on rat CG-4 (**a**) and human primary (**b**) OPCs. No antibody control is shown (grey). PI-88 treatment (100 μg/mL) resulted in a clear increase in OPC sulfation in a time-dependent manner. **c**, **d** Western blots for active β-catenin (αABC) and phosphorylated Smad 1/5 (p-Smad) following treatment of rat CG-4 OPCs treated with BMP7, WNT3a (both 50 ng/mL), and/or PI-88 (100 μg/mL). **e**, **f** Dose–response curves for WNT and BMP reporter activity in the presence/absence of PI-88 (20 h following treatment, *n* = 3, one-way ANOVA). ** and *** indicate significant effect of PI-88 by Dunnett’s post-test *p* < 0.01 and <0.001, respectively. **g** human primary OPCs were infected with lentiviral TCF reporter and treated with PI-88 (2 µg/mL) and/or WNT3a (50 ng/mL) (*n* = 3 fetal human samples, normalized mean ± SEM). **h** similarly, hOPCs were infected with BMP response element-dependent reporter and treated with PI-88 and/or BMP7 *(n* = 3). **g**, **h** Two-way ANOVA revealed significant effects of WNT/BMP and PI-88 treatment, * and **** indicates Sidak’s post-test *p* < 0.05 and <0.001, respectively. **i**–**n** The effect of PI-88 treatment on hOPC differentiation was assessed in the context of inhibitory BMP treatment. hOPC were treated with BMP and/or PI-88 and O4^+^ oligodendrocyte (red) and GFAP^+^ astrocyte (green) differentiation assessed after removal of mitogens. Quantification of O4% (**m**, *n* = 5 fetal samples) and GFAP% (**n**, *n* = 3 fetal samples). Two-way RM ANOVA revealed significant effects of BMP and PI-88 on O4^+^ differentiation (main effect *p* < 0.05). Mean ± SEM is shown. Scale: 50 µm.

As sulfatases act in the extracellular space, they represent a pharmacologically relevant target for manipulation in demyelinating disease. PI-88, a highly sulfated structural mimetic of heparan sulfate acts as a non-cleavable substrate and inhibitor for sulfatases^38,39^ and is currently in clinical trials for cancer therapy. We first assessed basal HS sulfation on rat CG-4 and human primary OPCs by flow cytometry using RB4CD12 antibody that recognizes highly sulfated HS GAG motifs^40^ (Fig. 6a, b). Treatment with PI-88 caused a progressive and persistent enrichment of HS sulfation at 30 min and 24 h (Fig. 6b).

We next examined whether PI-88 via induced increased HS sulfation could inhibit WNT and BMP signaling in OPCs. In rat CG-4 cells, activated β-catenin, a downstream effector of canonical WNT signaling was substantially induced following WNT3a treatment (Fig. 6c). Importantly, PI-88 treatment effectively blocked the effect of WNT3a. Likewise, PI-88 attenuated BMP7-induced Smad 1/5 phosphorylation (phospho-Smad (p-Smad)) (Fig. 6d). Using luciferase-expressing lentiviral reporters of WNT and BMP signaling, we found PI-88 exhibited a clear dose-dependent effect and antagonized WNT and BMP signaling with half-maximal inhibitory concentrations of 0.38 and 0.89 μg/mL for WNT3a and BMP7, respectively (Fig. 6e, f).

Next, we explored the effects of PI-88 on human primary OPC signaling. Lentiviral reporter infected hOPCs were treated with BMP/WNT ligands and/or PI-88. BMP and WNT ligand treatment (50 ng/mL) induced a robust >15-fold increase in reporter activity (two-way ANOVA, *p* < 0.05) that was significantly attenuated by PI-88 (Fig. 6g, h). PI-88 treatment alone did not alter BMP or WNT-dependent luciferase activity. Interestingly, when we compared the effect of PI-88 treatment on low and high dose WNT/BMP-induced luciferase, we observed similarly reduced luciferase activity following PI-88 treatment (Supplementary Figure 8). This suggests that the effect of PI-88 is noncompetitive and is consistent with the inhibition of sulfatase rather than direct agonist binding or another receptor-mediated mechanism. Thus, we concluded that PI-88 was capable of inhibiting BMP and WNT signaling via sulfatase inhibition in both rat and hOPCs.

### PI-88 rescues the BMP7-mediated inhibition of hOPC differentiation in vitro

The inhibitory effects of PI-88 treatment on BMP-induced transcription suggested that PI-88-mediated inhibition of sulfatase activity could prevent BMP-mediated effects on OPC cell fate^37^. hOPCs were cultured following mitogen withdrawal to induce oligodendrocyte differentiation. As described above, BMP7 (5 ng/mL) blocked O4^+^ differentiation (*n* = 4 fetal samples, two-way repeated-measure ANOVA *p* = 0.0006) (Fig. 6m). Following BMP7 treatment, PI-88 treatment significantly increased O4^+^ differentiation (Holm–Sidak *p* = 0.035). Likewise, BMP7 induced GFAP^+^ astrocyte fate from hOPCs (Fig. 6n). Following PI-88 treatment, BMP7 was unable to significantly increase astrocyte differentiation. In the absence of BMP7, PI-88 did not significantly alter hOPC fate. Taken together, these results suggest that pharmacologically biasing hOPCs toward a state of elevated 6-O sulfation is phenotypically relevant, opposing the BMP7-mediated induction of astrocytic fate in vitro.

### PI-88 promotes oligodendrocyte differentiation and remyelination following focal demyelination

**Fig. 7:**
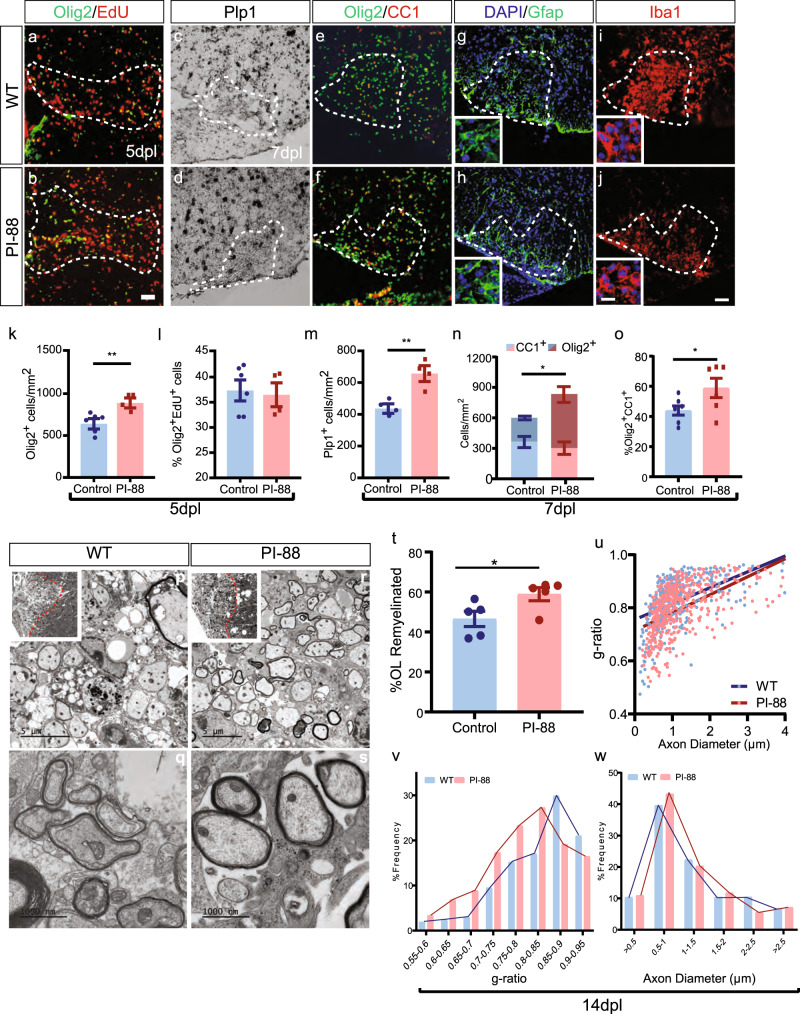
Sulfatase inhibitor PI-88 promotes OPC differentiation and remyelination following demyelination. PI-88 was injected into demyelinated lesions in adult mouse spinal cord at the time of lysolecithin injection. **a**, **b** Olig2^+^ recruitment and proliferation (EdU^+^Olig2^+^) was assess at 5 days post lesion (d.p.l.) by widefield epifluorescence (Olig2, green; EdU, red). **c**–**j** Oligodendrocyte lineage density (Olig2, green), differentiation (Plp1 and CC1, red), and the microglial/astrocyte response was assessed at 7 d.p.l. (Gfap, green; Iba1, red). Oligodendrocyte density and differentiation were assessed by *Plp1* in situ (**c**, **d**), and Olig2/CC1 immunofluorescence (**e**, **f**). **g**–**j** Astrocyte and microglial responses were assessed by Gfap and Iba1, respectively, and image inserts show higher magnification image to show morphology. **k**, **l** quantification of Olig2^+^ density (** indicates two-tailed *t* test *p* = 0.0079) and percentage of EdU^+^ cells among total Olig2 at 5 d.p.l. (*n* = 6 control, *n* = 4 for PI-88-treated mice). **m**–**o** At 7 d.p.l. quantification of *Plp1*^+^ cell density (**m**) (** indicates two-tailed *t* test *p* = 0.0048, *n* = 4 mice per group), Olig2^+^ and CC1^+^Olig2^+^ cell density (**n**) (* indicates total Olig2^+^ two-tailed *t* test *p* = 0.037), and percentage of CC1^+^ cells within the Olig2 population (**o**) (* indicates two-tailed *t* test *p* = 0.049, *n* = 6 mice for control, *n* = 5 mice for PI-88). **p**–**w** Analysis of remyelination by EM at 14 d.p.l. (*n* = 5 mice per group). **p**, **r** Inserts showing lesion location in ventrolateral white matter. **t** Proportion of remyelinated axons (* indicates two-sided *t* test *p* = 0.040) and **u** relationship between axon diameter and *g*-ratio (linear regression is shown). Frequency distribution of axonal *g*-ratio (**v**) and axon diameter (**w**) in lesion. *, ** indicate two-sided *t* test *p* < 0.05 and 0.01, respectively. Mean ± SEM is shown. Scale: 20 μm (**a**–**j**) and 5 μm (**p**–**r**) and 1000 nm (**q**–**s**).

As PI-88 was able to effectively modulate HS sulfation and antagonize WNT and BMP signaling in vitro, we next asked whether sulfatase inhibition by PI-88 could modulate remyelination. PI-88 (10 µg/ml) or saline was co-injected with lysolecithin. At 3 d.p.l., PI-88 did not influence lesion size, microglial infiltration, or astrogliosis. Loss of oligodendrocytes and OPCs within the lesion was likewise complete in both groups (Supplementary Figure 9). As such lesion formation was indistinguishable between PI-88-treated and -untreated animals. At 5 d.p.l., during OPC recruitment, we observed a significant >25% increase in Olig2^+^ density following PI-88 treatment (*n* = 6–7 mice, *t* test *p* = 0.008) (Fig. 7a, b, k) that was not due to increased proliferation (EdU%, *p* = 0.78) (Fig. 7l). Similar to our observations following *Sulf1/2* cKO, PI-88 treatment did not notably influence astrocytic or microglial responses at 7 d.p.l. (Fig. 7g, j and Supplementary Figure 10). At 7 d.p.l., the density of oligodendrocytes, defined as the density of either *Plp1*^+^ or CC1^+^Olig2^+^ cells, was significantly increased by PI-88 (*n* = 4, both *p* < 0.05) (Fig. 7m, n). Similar to *Sulf1/2* cKO, the overall Olig2^+^ oligodendrocyte lineage cell density was significantly increased by PI-88 treatment (*p* = 0.037) (Fig. 7n). Consistent with accelerated differentiation, the percentage of CC1^+^ oligodendrocytes among total Olig2^+^ was significantly increased by PI-88 (*n* = 6–7) (Fig. 7o). Thus, PI-88 treatment acts in a similar manner to *Sulf1/2* cKO resulting in increased oligodendrocyte density following demyelination.

Next, we examined whether enhanced differentiation following PI-88 treatment resulted in accelerated remyelination (Fig. 7p–s). We found that PI-88 treatment significantly increased the proportion of remyelinated axons at 14 d.p.l. (*n* = 5 mice per group, *t* test *p* = 0.040) (Fig. 7t). Linear regression analysis of axon diameter vs. *g*-ratios demonstrated a significant reduction of *g*-ratio following PI-88 treatment (Fig. 7u). Likewise, the distribution of *g*-ratios binned by axon diameter was left-shifted and indicated increased myelin sheath thickness in PI-88-treated mice (Fig. 7v). Similar to *Sulf1/2* cKO, PI-88 treatment did not induce axonal swelling confirmed by axon diameter frequency distribution (Fig. 7w). Together, these data demonstrate that PI-88 treatment promoted both OPC differentiation and accelerated remyelination following demyelination.

### PI-88-induced oligodendrocyte differentiation following differentiation is mediated via inhibition of sulfatase

**Fig. 8:**
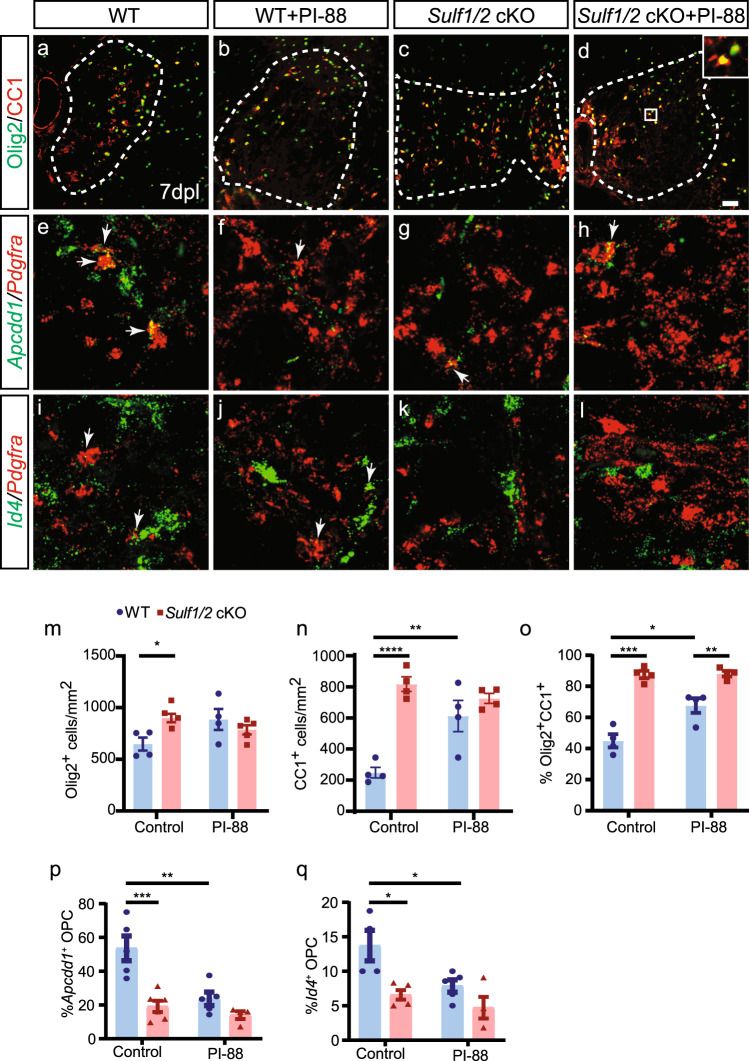
PI-88 treatment accelerated OL differentiation via inhibition of *Sulf1/2* following demyelination. Wild-type (WT) and *Sulf1/2* cKO mice underwent focal spinal cord demyelination with or without PI-88 injection and were sacrificed at 7 days post lesion. **a**–**d** Olig2 (green)/CC1 (red) immunofluorescence. **e**–**l** Confocal RNAscope in situ hybridization for OPC-expressed *Pdgfra* (red) and either WNT target gene *Apcdd1* (green, **e**–**h**) or BMP target gene *Id4* (green, **i**–**l**). Arrows indicate double-labeled OPCs. **m**–**o** Quantification of oligodendrocyte lineage density (Olig2^+^, **m**) (*n* = 4 mice for WT, *n* = 5 mice for *Sulf1/2* cKO groups), postmitotic CC1^+^Olig2^+^ cells (**n**) (*n* = 4 mice per group), and percentage of CC1^+^ cells within the Olig2 population (**o**) (*n* = 4 mice per group). Quantification of WNT pathway activity *Appcd1*% (**p**) (*n* = 5 mice for control WT and PI-88 treated WT, *n* = 6 for control *Sulf1/2* cKO, and *n* = 4 for PI-88 treated *Sulf1/2* cKO) and BMP pathway activity *Id4*% (**q**) (*n* = 4 mice for control WT and PI-88 treated *Sulf1/2* cKO groups and *n* = 5 for PI-88 treated WT and control *Sulf1/2* cKO groups) among *Pdgfra*^+^ OPCs. Mean ± SEM is shown. *, **, ***, and **** indicate two-way ANOVA Holm–Sidak post-test *p* < 0.05, <0.01, <0.001, and <0.0001, respectively. Scale: 20 µm.

To determine the mechanism by which PI-88 increased oligodendrocyte differentiation, and whether the effects of PI-88 were dependent on OPC-expressed sulfatase, we treated mice with PI-88 in the context of OPC-specific sulfatase deletion (i.e., *Sulf1/2* cKO). We induced lysolecithin lesions in *Sulf1/2* cKO and treated them with PI-88 (*n* = 4–5 mice per group) (Fig. 8). As described above, PI-88 treatment or *Sulf1/2* cKO alone significantly increased Olig2^+^ oligodendrocyte lineage density (Fig. 8m), Olig2^+^CC1^+^ density (Fig. 8n), and the proportion of CC1^+^ oligodendrocytes (Fig. 8n). Importantly, we did not observe an additive effect of PI-88 treatment on *Sulf1/2* cKO mice for these parameters (Fig. 8m–o). Two-way ANOVA revealed a highly significant interaction between genotype and PI-88 treatment on all parameters. Thus, PI-88 likely leads to increased oligodendrocyte differentiation primarily via inhibition of OPC-expressed *Sulf1/2*.

Finally, to determine if PI-88 might similarly regulate WNT and BMP signaling and exert a pro-differentiative effect via WNT/BMP antagonism, we examined the expression of WNT and BMP target genes following *Sulf1/2* cKO and/or PI-88 treatment using in situ hybridization (Fig. 8e–l). Similar to our results using pharmacological manipulation of WNT and BMP pathways, we observed significantly decreased expression of *Apcdd1* (WNT target) and *Id4* (BMP target) in *Pdgfra*^+^ OPCs in *Sulf1/2* cKO mice (two-way ANOVA, *Sulf1/2* cKO main effects *p* = 0.0003 and 0.0019 for *Apcdd1* and *Id4*, respectively, *n* = 4–6 mice per group) (Fig. 8p, q). Likewise, PI-88 treatment alone significantly inhibited expression of both genes in *Pdgfra*^+^ OPCs (two-way ANOVA, PI-88 effect *p* = 0.0021 and 0.0019 for *Apcdd1* and *Id4*, respectively). Combined treatment of PI-88 in *Sulf1/2* cKO mice did not significantly decrease WNT or BMP target gene expression any further compared to *Sulf1/2* cKO alone (Holm–Sidak, both *p* > 0.5). These observations suggest that PI-88 acts via OPC-expressed *Sulf1/2* to influence BMP and WNT signaling in OPCs and thereby promote myelin repair.

## Discussion

The inhibitory tissue environment of chronic demyelinated lesions acts to prevent efficient myelin repair and remyelination in MS^41^. The cellular environment and local ECM determine the availability and signaling of multiple growth factors and cytokine pathways. Here, we show that sulfation of HSPGs critically influences the paracrine signaling environment surrounding OPCs and can be modulated to improve recruitment and formation of new oligodendrocytes leading to accelerated myelin repair. These data demonstrate that extracellular sulfatases expressed by human and mouse OPCs promote inhibitory WNT and BMP signaling following demyelination and that ablation of sulfatase function either by conditional genetic deletion or pharmacological inhibition can enhance remyelination.

The mammalian genome contains two sulfatase genes that share substrate specificity but differ in expression pattern in a cell- and tissue-specific manner^15,16^. We found that OPCs express high levels of sulfatases that are eliminated during oligodendrocyte differentiation. During mouse CNS development, both sulfatases were expressed by OPCs and immature oligodendrocytes. In contrast, hOPCs express SULF2 >10^4^-fold higher than SULF1. The significance of this species difference in expression is unclear but has significant implications for the design of sulfatase-specific small molecules. In contrast, the similarities in terms of resultant phenotype following sulfatase deletion suggest an important conservation of function between human and mouse signaling. In the adult mouse CNS, Sulf1/2 expression is largely restricted to OPCs and a subpopulation of cortical neurons located in layers V and VI, as previously described 25 (Allen Brain Atlas). Neuronal Sulf2 expression suggests that the heparanome within cortical demyelinated lesions differs from subcortical white matter and may potentiate inhibitory signals that contribute to failed remyelination. Intriguingly, sulfatase expression remained predominantly restricted to OPCs following demyelination in both mouse and human lesions. While the specific HSPG core proteins that undergo sulfatase-mediated editing are unknown, syndecan-3 is noteworthy due to its high expression in fetal and adult hOPCs^21,37^ and in cultured murine cells^42^.

The role of heparanome sulfation has not been previously studied in the context of demyelination. Constitutive sulfatase expression by OPCs suggests these enzymes prevent complete 6-O sulfation and our functional studies indicate that increased HSPG sulfation is associated with inhibition of various extracellular signaling cascades. The resting state of OPC heparanome is predominately composed of highly sulfated HexA2S-GlcNS6S trisulfated disaccharide units that are specific among glial subtypes^22^. As such, sulfatases are ideally situated to modulate OPC signaling. The generation of highly sulfated HSPGs is catalyzed by sulfotransferases. These enzymes, expressed at high levels by OPCs, are localized to the Golgi apparatus and do not act once HSPGs are presented on the cell surface^43^. As such, the increased prevalence of surface HS sulfation following sulfatase inhibition is most likely due to de novo HSPG presentation and suggests a rapid turnover of OPC-expressed HSPGs.

In the adult CNS, sulfatase deletion in OPCs results in a substantial increase in recruitment and differentiation of oligodendrocyte lineage cells following demyelination leading to improved remyelination. Interestingly, antisense Sulf1 treatment during embryonic development results in reduced migration^44^. As HSPG sulfation is expected to influence multiple pathways, we observed distinct effects on OPC dynamics following demyelination. In addition to accelerated differentiation (assessed by density and proportion of postmitotic oligodendrocytes), we noted that sulfatase deletion resulted in progressive recruitment of Olig2^+^ cells such that the density of oligodendrocyte lineage cells progressively increased from 5 to 14 d.p.l. This contrasts with wt lesions that typically reach maximal density at 5 days. The mechanisms by which overall recruitment of OPC and subsequent generation of oligodendrocytes remain unclear and are likely complex due to the number of signaling pathways that may be influenced by sulfatase activity. As proof of concept, we assayed OPC migration in vitro following treatment with CXCL1, a known inhibitor of OPC migration and remyelination^45,46^. We found that CXCL1 treatment was able to reduce hOPC migration and, consistent with the role of HSPGs in CXCL1 signaling^47^, we found that SULF2 KD rescued the effect of CXCL1 on hOPC migration. Further, we found that WNT3A treatment reduces hOPC migration and that SULF2 KD was able to rescue this effect. These data suggest that OPC migration may be potentiated by *Sulf1/2* deletion following demyelination. Regardless, the effect of dual sulfatase deletion on oligodendrocyte number was striking as we observed a more 2-fold increase in oligodendrocyte number. Consistent with shared substrate specificity, the effect of individual sulfatase deletion was consistent with a model in which sulfatases act in a dose-dependent manner. As such, once a lower limit of sulfatase activity was reached, oligodendrocyte recruitment and differentiation were increased. The dose-dependent effects of sulfatase inhibition in vitro are consistent with this model and support the potential for future therapeutic intervention. Importantly, the effect of conditional deletion of *Sulf1/2* in NG2-expressing cells was restricted to OPCs as we did not observe pericyte Sulf1/2 expression either in normal CNS or following demyelination with similar results observed in purified hOPCs. As pericytes did not express Sulf1/2, conditional deletion of sulfatase in NG2-expressing cells resulted in the specific deletion of OPC-expressed sulfatase activity.

HSPG sulfation is known to regulate several signaling pathways^17^. Here, we show both WNT and BMP pathways are inhibited by sulfatase inhibition in OPCs. Canonical WNT signaling prevents efficient oligodendrocyte differentiation following demyelination^32,33^. WNT target genes are activated in OPCs following human white matter injury and correlate with failed differentiation and repair^48^. We found that endogenous sulfatases promote WNT signaling and sulfatase inhibition decreases WNT target gene expression in OPCs. This likely occurs in a ligand-receptor-dependent manner as TCF/LEF transcriptional activity was dependent on SULF2 expression in purified hOPCs.

The interpretation of the WNT activator and inhibitor treatment in the context of *Sulf1/2* cKO is somewhat complex. XAV939 treatment has been shown to promote OL differentiation via blockade of canonical WNT signaling^33^. We also observed that XAV939 was capable of promoting the oligodendrocyte differentiation following demyelination. In the context of *Sulf1/2* deletion, XAV939 was unable to further promote OL differentiation suggesting that *Sulf1/2* ablation may act in a similar manner to XAV939 to inhibit WNT signaling. Consistent with this, *Sulf1/2* cKO resulted in a significant downregulation of WNT target *Apccd1* following demyelination and SULF2 KD blocked WNT3A-mediated activation of a TCF/LEF reporter in hOPCs. A direct role of sulfatase and WNT signaling is further supported by the role of sulfatase in the regulation of HS sulfation and the sequestration of WNT by HSPG interactions^49^. On the other hand, CHIR-99021 was not able to rescue the effects of *Sulf1/2* cKO. CHIR-99021 acts as a GSK-3β inhibitor to potentiate canonical WNT signaling and would be expected to prevent oligodendrocyte differentiation if *Sulf1/2* acted solely via antagonism of WNT ligand/receptor interactions. The observation that *Sulf1/2* cKO is still able to promote differentiation and repair following CHIR-99021 treatment suggests that sulfatases in parallel and independently of WNT act on other pathways to regulate oligodendrocyte differentiation. One possible model to reconcile these differences is suggested by in vitro data in rodent OPCs showing that the effects of WNT ligands are dependent on secondarily inducing BMP release and signaling to modulate differentiation^50^. Our results in vivo would seem to fit this model, as WNT activation via CHIR-99021 would lead to increased BMP tone, which in turn would be antagonized in the *Sulf1/2* cKO. Alternate models are also possible and further investigation will be needed to test this intriguing possibility.

Our data support a clear role for sulfatase-mediated potentiation of BMP signaling following demyelination. Genetic sulfatase deletion in OPCs resulted in impaired BRE-dependent transcription and reduced Id4 expression by OPCs in vivo. BMP signaling is upregulated following demyelination^31,34^ and is active in MS lesions^30^. We found that BMP receptor antagonist increased both Olig2 recruitment and oligodendrocyte differentiation, as shown^51^. Sulfatase deletion in the context of BMP blockade did not further increase differentiation, suggesting that this process is dependent on BMP signaling. In contrast, intracellular activation of BMP signaling by treatment with A01, a Smurf1 E3 ligase-specific antagonist, could not be effectively compensated for by sulfatase deletion. Given the intracellular mode of action for A01, this is entirely consistent with a model in which HSPG sulfation regulations BMP ligand/receptor accessibility.

In addition to WNT and BMP, modulation of HSPG sulfation by sulfatases regulates several additional signaling cascades that influence the demyelinated lesion microenvironment. Previously, Sulf activity has been shown to potentiate PDGFαR signaling in glioblastoma^29^. PDGF is the principle OPC mitogen during development^37,52–54^, but does not appear to be rate-limiting following murine spinal cord demyelination^55,56^. In addition to PDGF, previous studies suggest that sulfatase activity inhibits FGF2 signaling^35,57–60^ and pharmacologically reduced sulfation blocks FGF responsiveness in OPCs^61,62^. As FGFR ablation in oligodendrocyte lineage cells results in hypomyelination^63^, inhibition of sulfatase leading to increased FGF signaling is consistent with our observed acceleration of remyelination and increased myelin sheath thickness following *Sulf1/2* cKO. In addition to PDGF and FGF2, heparan sulfatase regulation of inflammatory signaling cascades such as interferon-γ has been described^64^. As such, HSPG 6-O sulfation via regulation by OPC-expressed sulfatases provide the basis for the coordinated regulation of the lesion microenvironment.

Given that sulfatases are secreted into the extracellular milieu, it is likely that paracrine effects of sulfatase inhibition may influence other glial and inflammatory cell signaling following demyelination. Although a broad assessment of microglial numbers via Iba1 immunoreactivity and astrogliosis via Gfap did not indicate a major effect following sulfatase deletion, we cannot exclude the possibility that paracrine effects may influence the infiltration, proliferation, or activation of microglia and other immune cells within the lesion environment. Importantly, while lysolecithin-induced demyelination provides the ideal model to assess the effects of sulfatase deletion on the cellular dynamics of remyelination, paracrine influences of sulfatase inhibition cannot be ruled out due to the relative absence of adaptive immune system-related signaling in this model.

PI-88 is a heparin mimetic acting as a competitive antagonist to sulfatases^38^. The effects of PI-88 treatment on OPC dynamics following lysolecithin demyelination essentially phenocopied that of OPC-specific sulfatase deletion. Importantly, when PI-88 treatment was combined with *Sulf1/2* cKO, there were no additive effects and PI-88 treatment did not further influence OPC dynamics or differentiation. This is consistent with the effects of PI-88 being mediated solely via inhibition of OPC-expressed sulfatase. However, we cannot exclude the possibility that heparanase antagonism by PI-88 could influence the lesion environment^65^.

By modulating the local OPC niche in the context of demyelination, we have found a single target capable of modulating several signaling cascades in concert. Previous remyelination approaches have targeted single signaling pathways such as LINGO-1^66^, WNT^33^, RXRγ, and muscarinic receptor antagonists^20,67,68^, amongst others. Other approaches such as miconazole or clobetasol have less defined mechanisms of action^69^. While these and other small molecules have been discovered that promote OPC differentiation and remyelination in rodent models, given the apparent diversity of inhibitory cascades involved, it seems unlikely that targeting single pathways will yield an effective clinical intervention. As such, PI-88 may allow simultaneous modulation of several distinct signaling inputs in favor of a single biological goal, a unique approach to the treatment of demyelinating disease. In addition, as cell therapies for myelin disorders approach clinical translation^70^, modulation of the heparanome might represent a viable approach to overcome the local inhibitory environment into which cells are engrafted. Future studies will need to directly address the clinical utility of PI-88, and whether PI-88 or another Sulf-targeting compound could function synergistically with one or more of the previously described compounds to exact efficient myelin repair. Systemic PI-88 (Muparfostat) is currently being investigated in various oncology-related clinical trials (ClinicalTrials.gov), with a favorable safety profile reported^71^.

Taken together, OPC-expressed sulfatases by regulating their local heparanome, potentiate inhibitory signals present in demyelinating lesions that prevent efficient myelin repair. Treatment with PI-88 represented a potent means to inhibit sulfatases and support the potential for therapeutic approaches aimed at sulfatase inhibition. CNS optimized delivery of PI-88 or similar heparan mimetics may represent a potent and multifaceted approach to achieve enhancement of endogenous myelin repair in diseases such as MS.

## Methods

### Human CD140a/PDGFαR cell and tissue preparation

Fetal brain tissue samples, between 17 and 22 weeks of gestational age, were obtained from Advanced Bioscience Resources (Alameda, CA) with informed consent obtained from all donors. Following review by the University at Buffalo Research Subjects Institutional Review Board, the tissue acquisition and research were not deemed to involve human subjects as defined under HHS regulations 45 CFR 46, 102 (f). Forebrain samples were minced and dissociated using papain and DNase as previously described^72^. Magnetic sorting of CD140a/PDGFαR-positive cells was performed as described^73^. hOPCs were maintained on plates coated with poly-ornithine and laminin, in human neural differentiation media supplemented with 20 ng/mL PDGF-AA (PeproTech) and 5 ng/mL NT-3 (PeproTech)^20^. Fetal tissue was fixed in 4% paraformaldehyde (PFA) and cryoprotected in a sucrose gradient (7.5% sucrose overnight, followed by 15% sucrose overnight), and frozen in OCT medium (Tissue-Tek). Serial 16 µm transverse sections were cut using a Leica cryostat and stored at −80 °C.

### RNA extraction and RT-PCR analysis

Total RNA was isolated from hOPCs following mitogen withdrawal and real-time RT-PCR performed as described in ref. ^18^. Briefly, mRNA was isolated using an E.Z.N.A Total RNA Kit I (Omega Bio-Tek, Norcross, GA) according to the manufacturer’s protocols and complementary DNA prepared (SuperScript III Kit; Invitrogen, Carlsbad, CA). Human-specific primers for SYBR green-based PCR were designed using Primer Express (v1, Applied Biosystems, Foster City, CA). Primer sequences are shown in Supplementary Table 2. Gene expression was calculated by normalizing to glyceraldehyde 3-phosphate dehydrogenase and performing ΔΔCt analysis. Statistical significance was tested on log 2-transformed data using repeated-measures one-way ANOVA followed by Tukey’s post-test.

### Cloning and lentiviral preparation

pLVTHM vectors containing shRNA targeting SULF2 (GGAGTGGTGGTGTCAATA) or a non-targeted scrambled control (AACAGTCGCGTTTGCGACTGG) were used as previously described^13^. The pBARLHyg lentiviral reporter plasmid was a gift from Dr. Randall Moon (University of Washington)^74^. pGL3 BRE luciferase was a gift from Martine Roussel and Peter ten Dijke (Addgene, plasmid # 45126)^75^. The lentiviral pTRIP-EF1a backbone was a gift from Abdel Benraiss (University of Rochester, Rochester, NY)^76^. To generate L-BRE-Luc, pGL3 BRE Luciferase was digested with *Kpn*I (NEB), blunted using a commercial blunting kit (NEB), and digested with *Xba*I (NEB). The pTRIP-Ef1α plasmid was linearized by digestion with *Mlu*I (NEB), blunted, and digested with *Xba*I to remove Ef1α-mCherry-WPRE. The BRE-Luc fragment and the pTRIP-EF1a backbone were purified by gel extraction (Qiagen) and ligated with T4 DNA ligase (Invitrogen) to generate L-BRE-Luc. pBARLHyg, L-BRE-Luc and pLVTHM were packaged in lentivirus as previously described^52^. Briefly, HEK 293T cells were triple transfected with viral vector and packaging plasmids pLP/VSVG (Invitrogen) and psPAX2 (AddGene), and viral supernatant was collected 42 h later. pLVTHM viral titer was determined by quantification of pLVTHM-driven GFP expression.

### Oligodendrocyte differentiation and immunocytochemistry

For KD studies, hOPCs were seeded at 5 × 10^4^ cells/ml and transduced with the indicated lentivirus after 24 h. To initiate differentiation, PDGF-AA and NT-3 growth factors were removed, and cells cultured in ND media supplemented with human BMP7 (PeproTech), human WNT3a (R&D Systems) and/or PI-88 (gift of Medigen Biotechnology Corp., Taiwan), as designated, with media replenished after 48 h. Four days following growth factor removal, cells were live stained with O4 IgM hybridoma supernatant (gift of Dr. James Goldman, Columbia University), fixed in 4% PFA, and immunostained. For assessment of SULF2 expression, hOPCs were maintained as progenitors, and fixed and stained for SULF2 (Abcam, ab113405, 1:500). Where indicated, brefeldin A (Cell Signaling Technology) was added to hOPCs at 5 µg/ml for 5 h prior to fixation. Alexa 488-, 594-, and 647-conjugated secondary antibodies (Invitrogen) were used at 1:500 dilutions. Differentiation was quantified as the proportion of stained cells in ten random fields at ×10 magnification (using an Olympus IX70 microscope), representative of over 1000 cells total in each condition.

### Assessment of hOPC HS sulfation by flow cytometry

hOPCs were cultured as previously described, trypsinized, and collected in 0.02% EDTA/phosphate-buffered saline (PBS). Cells were resuspended in HS3A8V (RB4CD12) phage display antibody (1:10, gift from Toin van Kuppevelt, Nijmegen Medical Center, Nijmegen, The Netherlands). The His-tagged phage display antibody was detected by immunostaining with 6×-His Tag primary antibody (1:700, Abcam) and 1:500 Alexa 488-conjugated secondary antibody. All antibody incubations were for 30 min at +4 °C. Flow cytometry was performed using a BD Fortessa flow cytometer. Dead cells were excluded by forward scatter- and side scatter-based gating, and doublet discrimination was performed. Fluorescence intensity (FITC-A) was quantified from ~10,000 cells in each replicate, and data were normalized to peak fluorescence to facilitate presentation.

### Cell and secreted protein isolation and western blot

For cellular and secreted SULF2 expression analysis, hOPCs were cultured for 24 h in ND media supplemented with 20 ng/mL PDGF-AA and 5 ng/mL NT-3. For oligodendrocyte SULF2 expression, PDGF-AA and NT-3 were removed to allow oligodendrocyte differentiation for 3 days. Supernatants were collected, 0.22 μm filter sterilized, and precipitated with acetone. Cells were washed in PBS, lysed, and sonicated in a buffer containing 50 mM β-glycerophosphate, 20 mM HEPES buffer, 1% Triton X-100, protease inhibitors (Roche), phosphatase inhibitor cocktail 2 (Sigma), and phosphatase inhibitor cocktail 3 (Sigma). For WNT and BMP pathway activity analysis, CG-4 rat OPCs were cultured on poly-d-lysine-coated flasks in ND media supplemented with 10 ng/mL PDGF-AA and 10 ng/mL bFGF (PeproTech). Cells were treated with 50 ng/mL human WNT3a, 50 ng/mL human BMP7, and/or 100 μg/mL PI-88 for the indicated time-points, washed in PBS, lysed, and sonicated as above. Protein concentrations were determined using a Bradford assay (Bio-Rad), and 30 µg protein was used for slot blot of secreted protein or added onto 10% polyacrylamide gels for electrophoresis and western blot. Proteins were transferred to nitrocellulose membranes and blocked with commercial blocking buffer (Rockland) or 5% milk and 0.1% Tween-20 (Calbiochem) in PBS depending on the primary antibody utilized. Primary antibodies against SULF2 (1:500, Abcam), p-SMAD 1/5 (1:1000, CST), active β-catenin (1:2000, Millipore), and β-actin (1:10,000, CST) were applied overnight at 4 °C in blocking buffer. IR680RD and IR800CW secondary antibodies (LI-COR) were diluted 1:5000 in Rockland blocking buffer. Blots were imaged using the LI-COR Odyssey Infrared Imaging System. Examples of uncropped and unprocessed scans are provided in Source data.

### Migration assay

hOPCs maintained in PDGF-AA and NT-3 were infected with either SULF2 KD or control scrambled lentivirus (1 multiplicity of infection). After 48 h, cells were seeded onto the upper surface of laminin-coasted transwell membranes (5 × 10^3^/insert; 8-µm pore diameter, VWR). Recombinant human WNT3a (R&D Systems), PDGF-AA (Peprotech), or the combination of PDGF-AA and CXCL1 (R&D Systems) were added to the lower chamber (WNT3a, 5 ng/ml; PDGF-AA, 10 ng/ml; CXCL1, 5 ng/ml). At 16 h, cultures were fixed with 4% PFA and stained with DAPI (4′,6-diamidino-2-phenylindole). Cells on the upper surface of the transwell were removed and the remaining migrant cells were imaged (four ×10 fields/well; Olympus IX83) and counted in an unbiased manner using a Python *OpenCV*-based approach. Two-way ANOVA with Tukey’s HSD post hoc statistics were performed in R.

### Luciferase assays

OPCs were seeded at a density of 2.5 × 10^4^ cells/ml and maintained as progenitors in ND media supplemented with PDGF-AA and NT-3. One day post seed, cells were infected with lentiviral luciferase reporter constructs for 24 h, after which media were changed to fresh ND media supplemented with growth factors, BMP7, WNT, and/or PI-88, as indicated. Twenty hours post treatment, luminescence response was quantified using the Promega Bright-Glo reagent and a Bio-Tek plate reader, in accordance with the manufacturer’s recommendations. Background luminescence was subtracted from all measurements and luminescence readings were normalized to the average value of the untreated control in each experiment. Data are presented as the mean ± SEM per individual human sample (three technical replicates).

### Animals and surgery

All experiments were performed according to protocols approved by the University at Buffalo’s Institutional Animal Care and Use Committee. NG2creER:Rosa26YFP animals were a gift from Akiko Nishiyama (University of Connecticut, Storrs, CT)^26^. *Sulf1/2* double-floxed (*Sulf1*^fl/fl^*Sulf2*^fl/fl^) animals were a gift from Dr. Xinping Yue (LSU Health Sciences Center, New Orleans, LA)^27^. *Sulf1/2* double-floxed (*Sulf1*^fl/fl^*Sulf2*^fl/fl^) animals were crossed with the NG2creER; RosaYFP mice to generate three colonies NG2creER:YFP:Sulf1^fl/fI^, NG2creER:YFP:Sulf2^fl/fI^, and NG2creER:YFP:Sulf1/2^fl/fI^. Conditional *Sulf1*, *Sulf2*, and *Sulf1/2* KO by cre-mediated recombination in adult NG2^+^ OPCs were achieved by intraperitoneal administration of tamoxifen (200 mg/kg, Sigma) for 5 days, the last of which occurred 7 days prior to surgery. All experimental mice received tamoxifen in an identical manner and littermate controls lacking cre were compared with cre-containing cKO mice. For experiments using PI-88, 8–11-week-old female BalbC mice were purchased from Envigo.

Focal demyelination of the young adult (8–11 week) mouse spinal cord was induced as previously described^77^. Briefly, animals were anesthetized under isoflurane, and 0.5 μL of 1% lysolecithin (lα-lysophosphatidylcholine, Sigma) was directly injected into the dorsal and ventral funiculus of the spinal cord between two thoracolumbar vertebrae. To assess the role of BMP and WNT modulation in the context of *Sulf1/2* deletion, CHIR-99021 (3 µM, Axon MedChem), XAV939 (0.1 µM, Tocris), A01 (100 nM, Tocris), or LDN-193189 (100 nM, Tocris) or matched volume saline (1:50) were co-injected with 1% lysolecithin (wt/vol). For experiments involving PI-88, 10 µg/ml PI-88 was co-injected with 1% lysolecithin, and demyelination induced by injection of the mixture into the spinal cord. Groups of WT and *Sulf1/2* cKO animals were co-injected with lysolecithin and drug/vehicle.

### Animal tissue processing and analysis

Animals were euthanized at 3, 5, 7, or 14 d.p.l. by transcardial perfusion of saline, followed by 4% paraformaldehyde under deep anesthesia. Tissue processing and lesion identification were performed as described previously^77^. Slides immediately adjacent to the lesion centers, identified by solochrome cyanine staining, were used for all immunohistochemical procedures. Primary antibodies utilized were rabbit anti-Olig2 (1:500, Millipore), mouse anti-CC1 (1:50, Millipore), mouse anti-GFAP (1:300, Sigma), rabbit anti-Iba1 (1:300, Wako Chemicals USA), cleaved caspase-3 (Asp175) (1:250, Cell signaling), and rabbit anti-NG2 (1:200, Millipore). Alexa 488-, 594-, and 647-conjugated secondary antibodies (Invitrogen) were used at 1:500. In situ hybridization for Plp1 was performed as described in ref. ^77^. Higher magnification examples of CC1/Olig2 staining demonstrating positive cell criteria used for quantification are shown (Supplementary Figure 11). Images of spinal cords were captured at ×20 magnification by widefield epifluorescence using an Olympus IX51 with Prior stage and were stitched together (Fiji). For each marker, the average from at least two sections was quantified for each animal. Lesions with cross-sectional area <10,000 µm^2^ were excluded from the analysis, as were lesions that extended into surrounding gray matter. All quantification was performed by an investigator blinded to the identity of the samples.

### Electron microscopy

For assessment of remyelination, tissue was processed as described in Welliver et al. ^77^. Briefly, mice were sacrificed at 14 days post lesion by transcardial perfusion with 2% glutaraldehyde in 0.1 M phosphate buffer and spinal cords were extracted (*n* = 5 per group), 1-mm-thick blocks surrounding the spinal cord lesion were processed through osmium tetroxide, dehydrated through ascending ethanol washes, and embedded in TAAB resin (TAAB Laboratories). One micrometer section were cut, stained with 1% toluidine blue (Sigma-Aldrich), and examined by light microscopy to identify lesions. Selected blocks with lesions were trimmed, ultrathin sections cut, and examined by electron microscopy (Hitachi, HT7800). Images were acquired at ×2500 magnification. Analyses of remyelinated axons and *g*-ratios were performed blinded. For remyelination counts, a minimum of 800 axons was counted for each animal from at least six different fields, with all animals per treatment group. Analysis of *g*-ratio was performed as described in Dillenburg et al. ^78^. Briefly, axon and fiber diameters were measured using FIJI (diameter = 2 × √[area/*π*]). A minimum of 100 axons was analyzed per animal. The frequency distribution of axon diameter and *g*-ratio of remyelinated axons was calculated by binning on axon diameter and *g*-ratio.

### Human MS tissue

MS tissue was prepared as described in Tripathi et al. ^79^. Briefly, brains were collected as part of the tissue procurement program approved by the Cleveland Clinic Institutional Review Board. Informed consent was obtained from all tissue donors. Tissue specimens were not considered “human subjects” due to the absence of interaction with living patients and the use of autopsy materials under HHS regulations 45 CFR Part 46. Brains were removed according to a rapid autopsy protocol, sliced (1 cm thick), and then fixed in 4% paraformaldehyde and 30 µm sections were cut for morphological studies. Lesions were characterized for demyelination by immunostaining using proteolipid protein (PLP). Chronic active demyelinated lesions were analyzed from two patients diagnosed with secondary progressive MS (53–57 years old, disease duration >10 years, postmortem interval <8 h).

### Fluorescent in situ hybridization (FISH)

Double label FISH was performed with probes targeting Sulf2 (GenBank: NM_001252578.1), Sulf1 (GenBank: NM_ 001198565.1), Plp1 (GenBank: NM_ 000533.4), Pdgfra1 (GenBank: NM_011058.2), Ki67 (GenBank: NM_001081117.2), Apcdd1 (GenBank: NM_133037.3), and ID4 (GenBank: NM_031166.2) mRNA by using the RNAscope Fluorescent Multiplex Detection Kit (Advanced Cell Diagnostics) according to the manufacturer’s instructions. Postnatal days 7 and 28 and 24-week-old mice were euthanized and perfused with 0.9% saline, followed by 4% paraformaldehyde. Dissected mice brains and spinal cords were cryopreserved in a sucrose gradient (10, 20, and 30%) for 24 h and were snap frozen in OCT and sectioned coronally at 16 µm thickness. Fixed sections were baked at 60 °C for 1 h, washed with ethanol, followed by tissue pretreatment, probe hybridization following RNAscope fluorescence multiplex assay, and sections were counterstained with DAPI to visualize nuclei. Positive signals were identified as punctate dots present in the nucleus and cytoplasm. All tissues were tested using negative control probes (ACD Bio) to control for nonspecific binding (Supplementary Figure 2a). For Sulf1 and Sulf2 probes, the expression patterns were verified by comparison with known sulfatase-specific patterns of neuronal expression in cortical layer V (Sulf2)^25^ and layer VI (Sulf1) (Allen Brain Atlas) (Supplementary Figure **2**b–f). For all RNAscope-based imaging, confocal Z stack images with optical thickness 0.2 µm were taken and stacked images are shown (Zeiss LSM 510 Meta confocal).

### Statistics and reproducibility

All quantification and data analysis were performed by an investigator blinded to the identity of the samples. All statistical analyses were performed using GraphPad Prism (San Diego, CA). Data were compared by Student’s *t* test, one-way ANOVA, or two-way ANOVA, where appropriate; significance was considered at *p* < 0.05. Exact *p* values and details of each statistical test are available in the accompanying Source data file. With the exception of MS tissue (*n* = 2), representative micrographs are shown from experiments performed with at least three or more biologically independent replicates. For experiments in which more than three biologically independent replicates are included, the number of replicates is provided in the relevant figure legends and available in Source data. Data are reported as mean ± SEM.

### Reporting summary

Further information on research design is available in the Nature Research Reporting Summary linked to this article.

## Supplementary information

Supplementary Information

Reporting summary

## Data Availability

A reporting summary for this article is available as Supplementary information file. The main data supporting the findings of this study are available within the article and its Supplementary Figures. Complete statistics and exact *P* values for each test and post hoc comparison are also included within the source data. Additional details on datasets and protocols that support the findings of this study will be made available by the corresponding author upon reasonable request. Source data are provided with this paper.

## Source data

Source Data
