# Supplementary material for: Overcoming the inhibitory microenvironment surrounding oligodendrocyte progenitor cells following experimental demyelination
Source: Nat Commun. 2021 Mar 26;12:1923. doi: 10.1038/s41467-021-22263-4 (PMC7998003; doi:10.1038/s41467-021-22263-4)
Supplement: Supplementary file 1 — Supplementary Information [file 41467_2021_22263_MOESM1_ESM.pdf]

Supplementary Figures

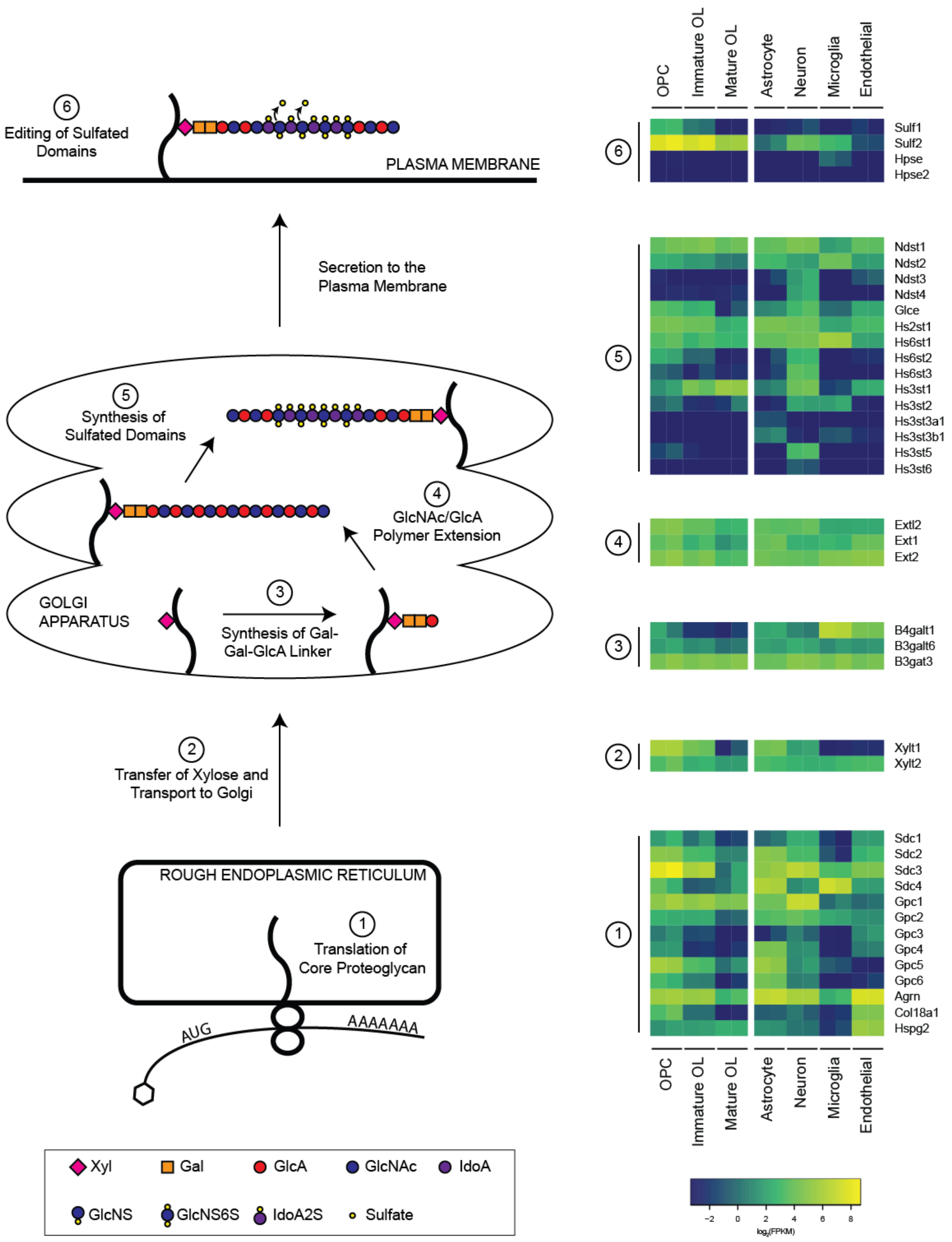

Supplementary Figure 1 (related to Figure 1a). Expression of HS-associated genes in the rodent CNS. The HS biosynthetic pathway is presented as a series of six biological steps beginning with translation of core proteoglycan mRNA in the rough endoplasmic reticulum and concluding with editing of HS side chains at plasma membrane [1-3]. HS-associated genes have been classified based on the involvement of their protein products in one of these six biosynthetic steps. The heat map presents gene expression on a log(FPKM) scale across murine purified CNS cell populations (Zhang et al., 2014).

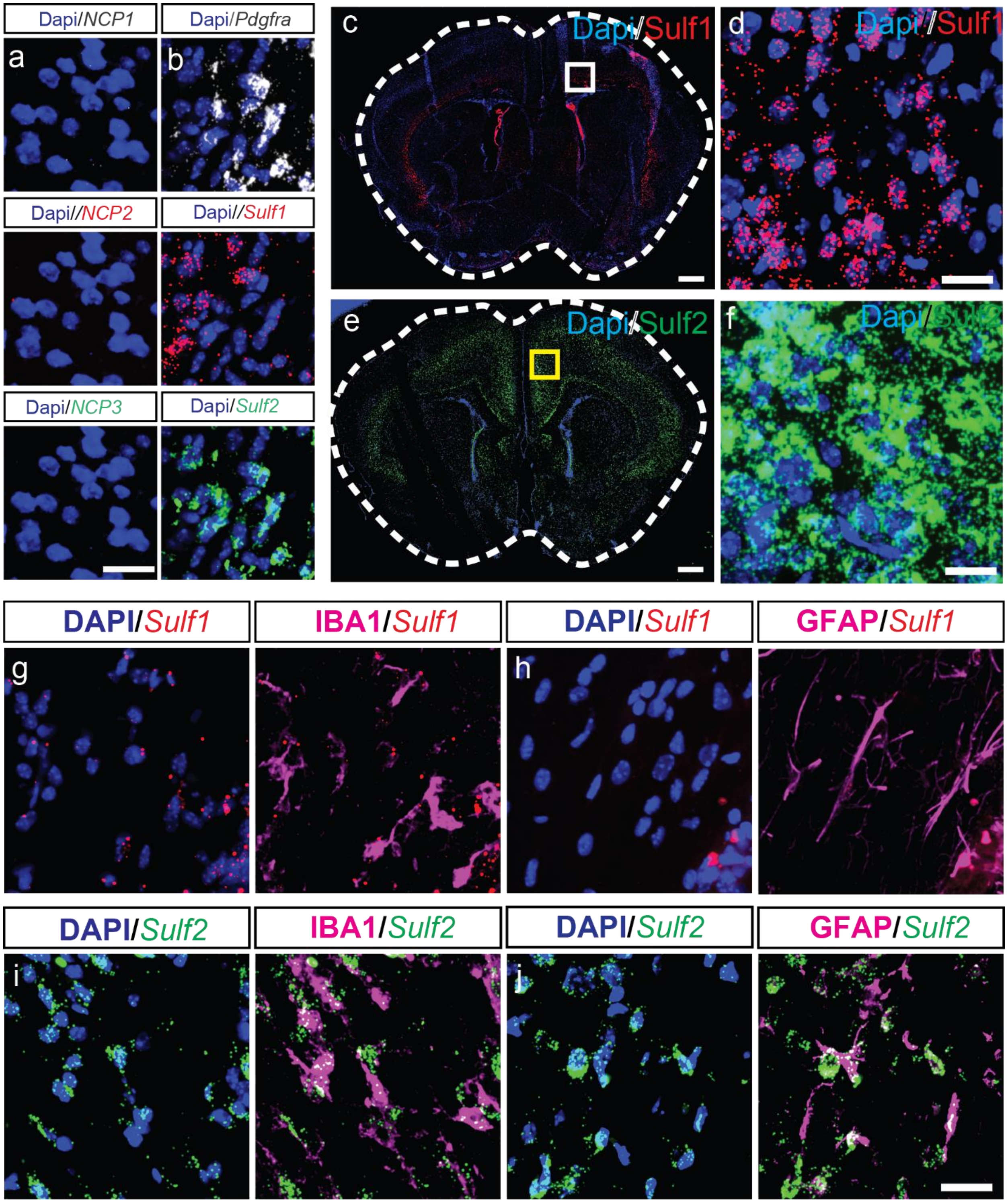

Supplementary Figure 2 (related to Figure 1). Sulf1 and 2 expression in postnatal day 7 mouse grey matter and white matter. a, RNAscope *in situ* hybridization (ISH)) of negative control probe 1, 2 and 3 demonstrating the lack of specific binding in mouse brain. **b,** inclusion of *Pdgfra*, *Sulf1* or *Sulf2* probe results in specific and distinct patterns of expression in neonatal white matter. **c-f,** *Sulf1* and *Sulf2* mRNA transcripts are abundant in developing cortical layer VI and V neurons, respectively, and correspond to previously described patterns of expression [4](Allen Brain Atlas). **g-j,** RNAscope *in situ* hybridization (ISH) and immunohistochemistry (IHC) of P7 mice brain revealed *Sulf2* expression by a subset of Gfap$^+$ astrocytes and Iba1$^+$ microglia in white matter (both by immunohistochemistry). In contrast, Sulf1 mRNA was not colocalized with Iba1 or and Gfap positive cells. Scale: **a-b,** 20 µm, **c-d**, 100 µm (a) and 20 µm (e-j).

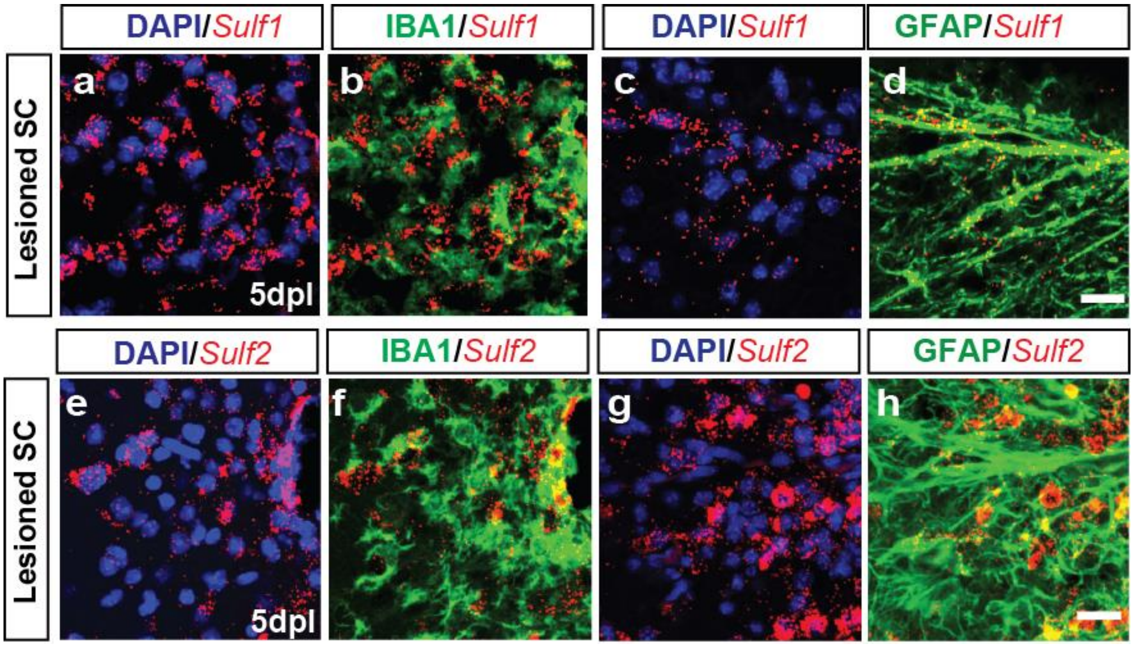

Supplementary Figure 3 (related to Figure 2). Increased Sulf2 expression in other glial cells after demyelination in lesion area. RNAscope *in situ* hybridization (ISH) and immunohistochemistry (IHC) of mice spinal cord after demyelination revealed *Sulf 1* and *2* expression by astrocytes and microglial in lesions as assessed by immunohistochemistry of Gfap and Iba1 and *Sulf1* and *2* mRNA transcripts. Scale: 20 µm. dpl: days post lesion.

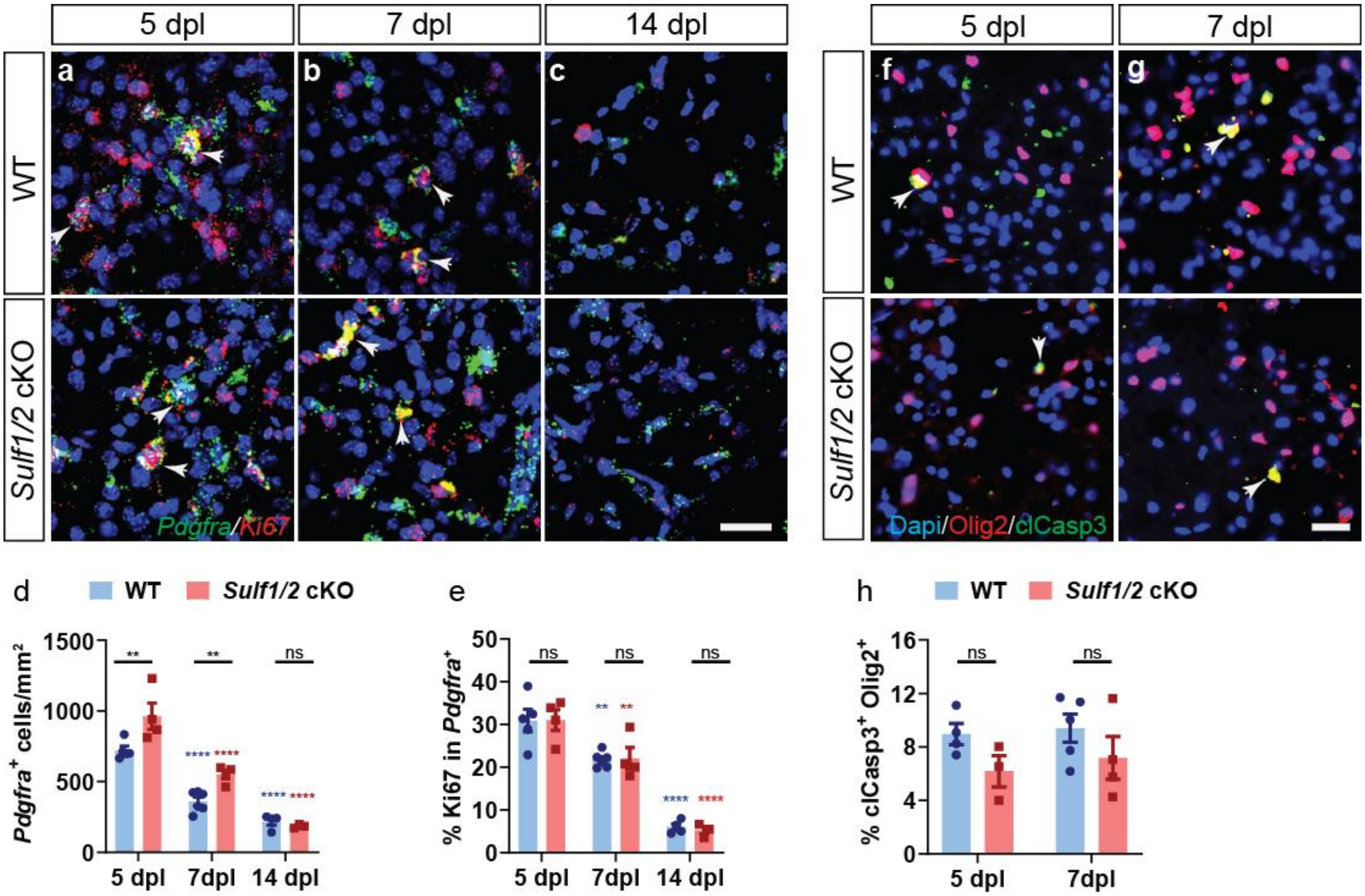

Supplementary Figure 4 (related to Figure 2). Conditional ablation of *Sulf1/2* accelerates recruitment of OPCs following demyelination. Tamoxifen-dependent OPC-specific ablation of both *Sulf1/2* in NG2$^+$ OPCs was initiated prior to demyelination. Control animals (lacking cre) were treated in an identical manner. **a-c,** *Pdgfra*$^+$ cell density and proliferation (Ki67$^+$Pdgfra$^+$) was assessed at 5, 7, and 14 days post-lesion (dpl) by dual RNAscope *in situ* hybridization (ISH). *Pdgfra* (green), *Ki67* (red) and DAPI (blue). White arrows denote *Pdgfra*$^+$ OPCs that co-expresses *Ki67*. The density (cells/mm^2) of Pdgfra$^+$ oligodendrocytes lineage cells (**d**) and percentage of Pdgfra$^+$Ki67$^+$ proliferating OPCs (**e**) was quantified (n = 5, 4, 6, 4, 4, 3 mice per group, left to right, for 5, 7 and 14 dpl, in WT and *Sulf1/2* cKO mice respectively). Two-way ANOVA for different time points. *, **, ***, **** indicate Two-way ANOVA Holm-Sidak post-test $p < 0.05$, <0.01, <0.001, and < 0.0001 respectively. Blue denotes p-values compared to control wildtype while red denotes p-values compared to *Sulf1/2* cKO group. *Sulf1/2* ablation significantly increased OPC recruitment (**d**) at 5 and 7dpl compared to WT control. While there was no difference in the proportion of proliferating Ki67$^+$Pdgfra$^+$ cells compared to wild type at each time point. **f-g,** Cell death was assessed by cleaved caspase 3 colocalization (green) with Olig2$^+$ oligodendrocyte lineage cells (red). **h**, The percentage of Olig2$^+$clCasp3$^+$ apoptotic cells was quantified among total Olig2$^+$ oligodendrocyte lineage cells at 5 and 7 dpl (n = 4, 3, 5, 4 mice per group, left to right, for 5 and 7 dpl, in WT and *Sulf1/2* cKO mice respectively). There was no significant difference in the proportion of apoptotic clCasp3$^+$Olig2$^+$ cells compared to wild type at each time points. Mean ± SEM shown. Scale: 20 μm. dpl: days post lesion.

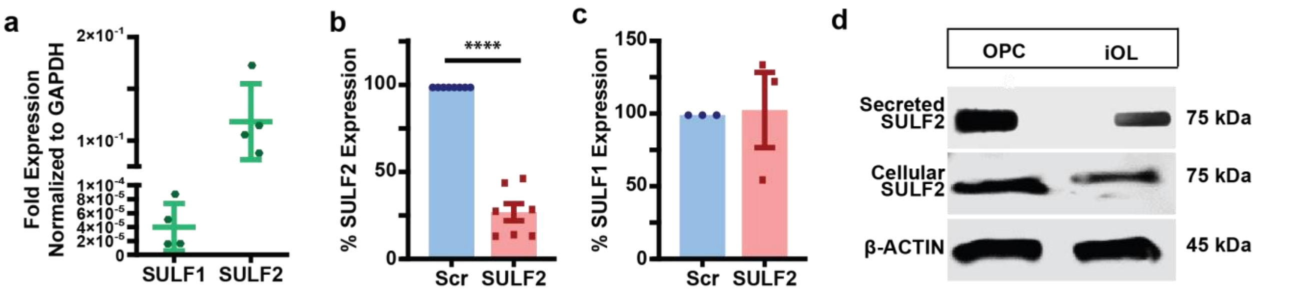

Supplementary Figure 5 (related to Figure 3). Lentiviral-mediated expression of SULF2-targeting shRNA reduces *SULF2* mRNA. Real-time quantitative PCR analysis of SULF1 and SULF2 expression in human OPCs (hOPCs). **a,** SULF1 and 2 expression in hOPC normalized to GAPDH (n = 4, human samples). **b,** hOPCs transduced with a lentivirus expressing shRNAi targeting SULF2 exhibit significantly reduced expression of SULF2 mRNA, compared to non-targeted scrambled (Scr) shRNA (**** p < 0.0001 one-sample two-tailed t-test, n = 8 human samples). **c**, SULF1 mRNA expression was not significantly affected by targeted SULF2 knockdown, compared to Scr controls (p = 0.90, one-sample two-tailed t-test, n = 3 human samples). Graphs represent mean ± SEM normalized to Scr. Analysis of SULF2 protein expression in hOPCs. **d**, Top panel: slot blot for SULF2 on conditioned media collected from cultured hOPCs in the presence or absence of growth factors for 3 days to initiate oligodendrocyte differentiation labeled as immature OL (iOL). Middle panel: western blot of matched hOPC lysates. 30µg protein was examined by slot blot and western blot using anti-Sulf2 antibody. Representative blot from two independent biological replicates. Mean ± SEM shown.

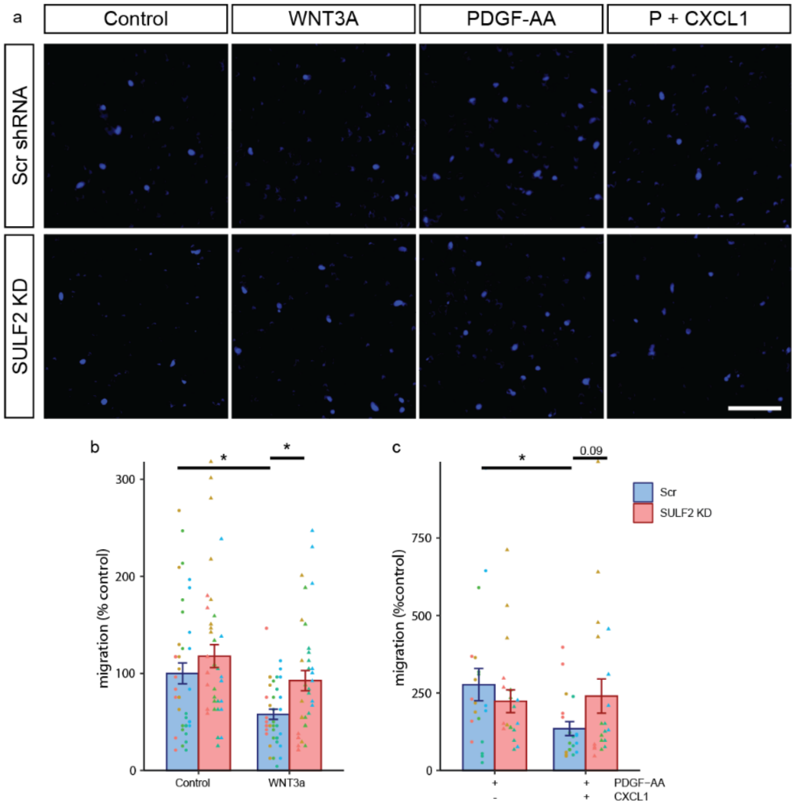

Supplementary Figure 6 (related to Figure 3). SULF2 regulates cytokine dependent hOPC migration. hOPC migration was assessed on transwell membranes following infection with lentivirus expressing SULF2 shRNA or a non-targeted scrambled shRNA (Scr). Migrant cells were imaged (**a**, DAPI) and quantified following treatment with either WNT3A (5ng / ml), PDGF-AA (10 ng/ml) or the combination of PDGF-AA (P) and CXCL1 (5 ng/ml). **b-c**, the number of migrant cells was assessed at 16 hours (mean ± SEM, from 5 fetal samples, two-way ANOVA, Tukey HSD post-test). WNT3A significantly impaired hOPC migration in Scr-infected cells (p = 0.0032) as did CXCL1 (Tukey HSD, p = 0.011), a known inhibitor of OPC migration [5] that is modulated by HSPG signaling [6]. Following SULF2 KD, both WNT3A and CXCL1 were ineffective and did not alter migration compared to untreated SULF2 KD hOPCs (Tukey HSD, p = 0.13 and 0.98, respectively). * p < 0.05 Tukey HSD. Linear model: nucleiCount ~ virus * condition * experiment. See source data for full statistics and linear model details. Mean ± SEM shown. Individual points from each biological replicate are shown in matching colors. Scale: 100 µm.

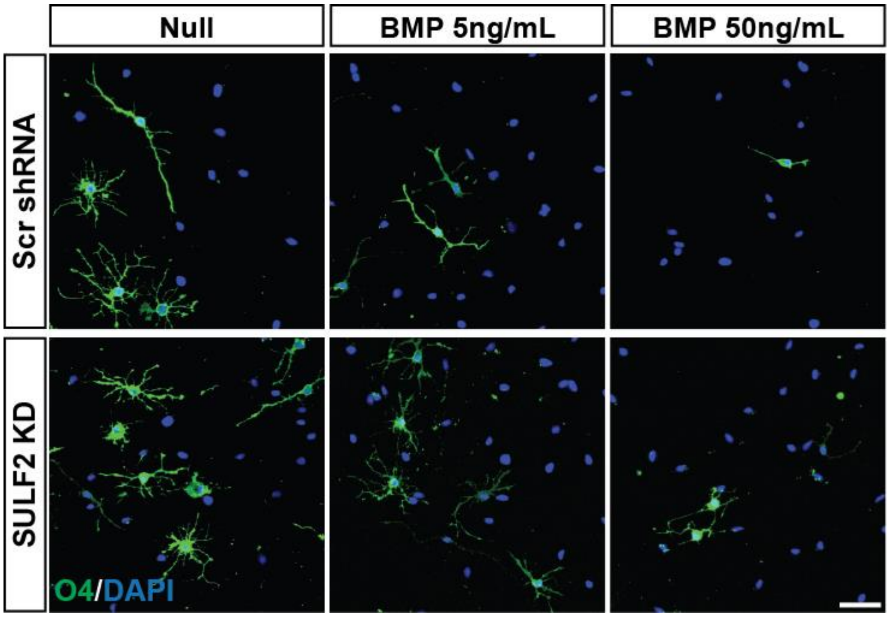

Supplementary Figure 7 (related for Figure 5). SULF2 knockdown increases differentiation despite inhibitory BMP signaling. Human OPCs were transduced with SULF2-targeted lentivirus or a scrambled control and allowed to differentiate in the absence or presence of BMP7, as indicated. After four days of differentiation, cultures were live-stained with differentiation marker O4 (green), and DAPI (blue) following fixation. Human OPC differentiation is significantly reduced following BMP7 treatment. SULF2 knockdown attenuated the effects of BMP, significantly increased differentiation of human OPCs, even in the absence of exogenous BMP, compared to scramble knockdown controls. Quantification shown in **Figure 5**. Scale: 100µm.

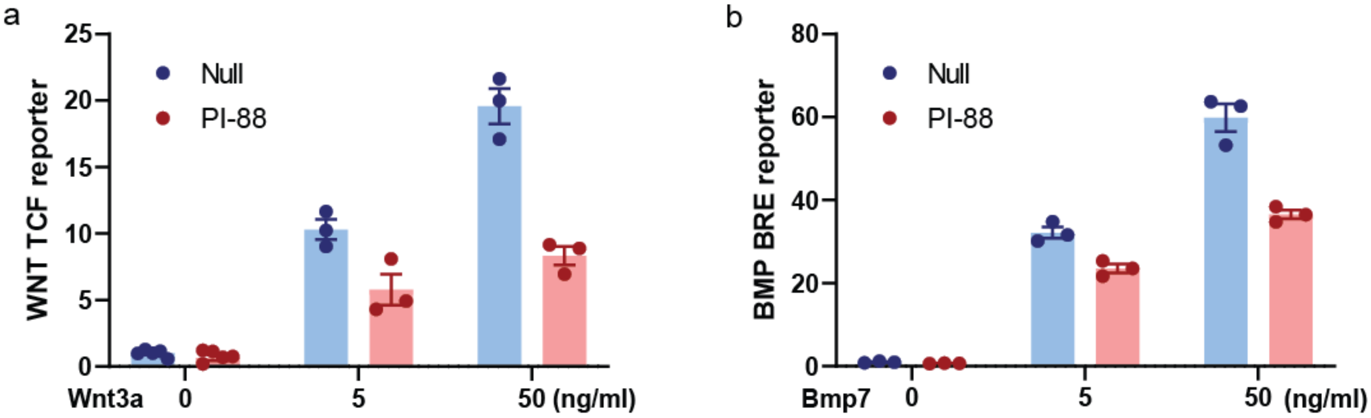

Supplementary Figure 8 (related to Figure 6). PI-88 inhibits WNT and BMP-related transcriptional activity in primary human CD140a-sorted OPCs. a, PI-88 (2 ug/ml) inhibits WNT-related transcriptional activity induced by WNT3a treatment (5 and 50ng/ml), as measured by the WNT TCF reporter activity. **b**, PI-88 (2 ug/ml) inhibits BMP-related transcriptional activity induced by BMP7 treatment (5 and 50 ng/ml), as measured by the BMP response element reporter activity. In all studies, human OPCs were infected with lentiviral luciferase reporters 24 hours prior to treatment with WNT3a or BMP7. Luciferase response was measured 20 hours post-treatment. Mean ± SEM shown (n = 3-4).

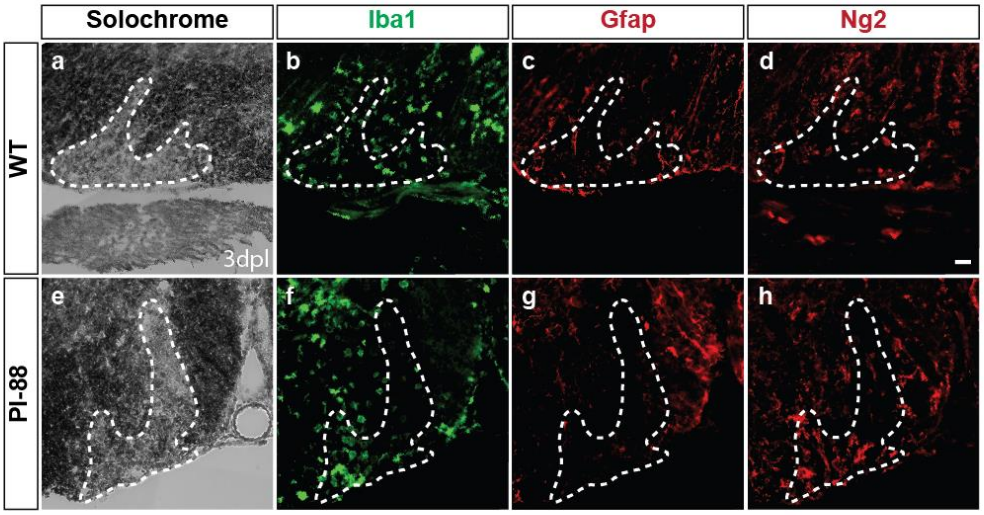

Supplementary Figure 9 (related to Figure 7). Administration of PI-88 does not alter lesion dynamics at 3 days post-lesion. Adult mice were subjected to lysolecithin-mediated focal demyelination of the spinal cord with or without simultaneous administration of 10µg/ml PI-88 directly into the lesion site. Animals were sacrificed at 3 days post-lesion (3dpl) to assess lesion dynamics. Demyelinated lesions of similar size were observed in the ventral white matter of mice (**a-e**) in both experimental groups. (**b-f**) Microglial infiltration, activation and proliferation were similar across experimental groups as assessed by Iba1 immunohistochemistry (green). (**c-g**) Gliosis was comparable in control and PI-88 treated animals, as assessed by Gfap immunohistochemistry (red). Oligodendrocyte progenitor cell infiltration was similar in control and experimental groups, as assessed by Ng2 immunohistochemistry (red) (**d-h**). n = 3-6 mice per group. Scale: 20 µm (**a-h**).

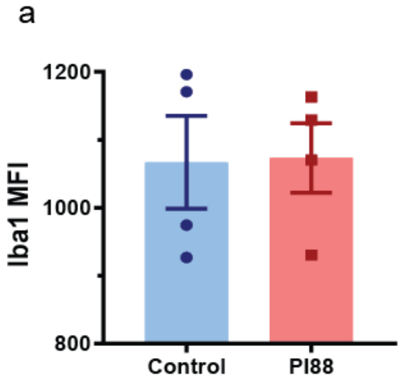

Supplementary Figure 10 (related to Figure 7). PI-88 treatment does not increase microglial infiltration in demyelinating lesions. PI-88 was injected into demyelinated lesions in young adult mouse spinal cord at the time of lysolecithin injection. **a.** Microglial responses was Iba1 immunofluorescence at 7 dpl (n = 4 mice per group, mean ± SEM).

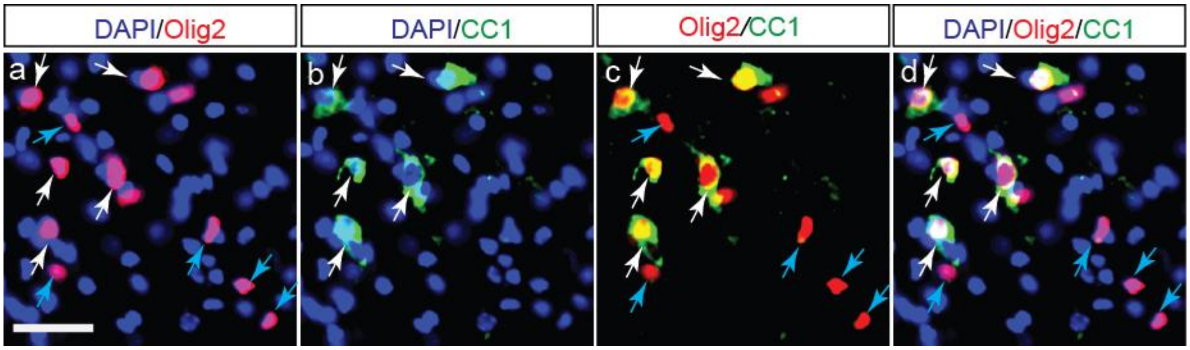

Supplementary Figure 11 (related to Methods). Method for quantifying mature oligodendrocytes. a-d. For identification and quantification of mature oligodendrocytes, we used Olig2/CC1 immunofluorescence and wide-field epifluorescence microscopy. $Olig2^+$ cells (red) with perinuclear $CC1^+$ (green) cells were considered as $Olig2^+CC1^+$ mature oligodendrocytes. White arrows indicate selection of $Olig2^+CC1^+$ oligodendrocytes (b-d). and blue arrow represent $Olig2^+CC1^-$ cells (c-d). Density of mature oligodendrocytes was calculated by counting cells within lesion border and the extent of differentiation assessed by measuring the percentage of $CC1^+$ cells within the Olig2 population. Scale: 60μm.

Supplementary Tables

Supplementary Table 1 (related to Figure 1) – Comparison of human and mouse HS-related gene expression.

Human Symbol	Description	Mouse OPC (FPKM)	Human OPC (FPKM)	Enrichment in OL lineage (Score)
**SULF1**	**Sulfatase 1**	**7.5 ± 0.4**	**0.2 ± 0.1**	**0.92**
**SULF2**	**Sulfatase 2**	**262.5 ± 53.0**	**99.9 ± 8.4**	**0.95**
**XYLT1**	**Xylosyltransferase I**	**63 ± 0.5**	**46.2 ± 7.3**	**0.78**
**HS3ST1**	**Heparan sulfate (glucosamine) 3-O-sulfotransferase 1**	**3.1 ± 0.8**	**ND**	**0.72 (very high in mature OL)**
**EXTL2**	**Exostoses (multiple)-like 2**	**26.4 ± 1.9**	**14.6 ± 1.2**	**0.57**
**CSPG4**	**Chondroitin sulfate proteoglycan 4**	**117.4 ± 15.5**	**88.0 ± 2.8**	**0.91**
**PDGFRA**	**PDGF receptor, alpha polypeptide**	**596.2 ± 32.4**	**422 ± 15.4**	**0.99**
**SOX10**	**SRY-box 10**	**147.1 ± 17.9**	**46.4 ± 0.9**	**0.99**

For mouse and human FPKM data, mean ± SEM shown, n=2. An oligodendrocyte lineage enrichment score was defined as the sum of expression (FPKM) across the three oligodendrocyte lineage populations (OPC, MOG, new OL) divided by the sum of its expression across all seven CNS populations. Mouse data taken from [7]. ND: not determined.

Supplementary Table 2 (related to Methods) – Real-time PCR primers.

Gene	Forward Primer	Reverse Primer
Human SULF1	ACCAGACAGCCTGTGAACAA	ATTCGAAGCTTGCCAGATGT
Human SULF2	TGAGGGAAGTCCGAGGTCAC	CTTGCGGAGTTTCTTCTTGC
Human GAPDH	GTGAAGGTCGGAGTCAACGG	CCTGGAAGATGGTGATGGGA

Supplementary References

1 Esko, J. D. & Selleck, S. B. Order out of chaos: assembly of ligand binding sites in heparan sulfate. *Annu Rev Biochem* **71**, 435-471, doi:10.1146/annurev.biochem.71.110601.135458 (2002).

2 Fernandez-Vega, I. *et al.* Specific genes involved in synthesis and editing of heparan sulfate proteoglycans show altered expression patterns in breast cancer. *BMC Cancer* **13**, 24, doi:10.1186/1471-2407-13-24 (2013).

3 Malfait, F. *et al.* Defective initiation of glycosaminoglycan synthesis due to B3GALT6 mutations causes a pleiotropic Ehlers-Danlos-syndrome-like connective tissue disorder. *Am J Hum Genet* **92**, 935-945, doi:10.1016/j.ajhg.2013.04.016 (2013).

4 Zeisel, A. *et al.* Brain structure. Cell types in the mouse cortex and hippocampus revealed by single-cell RNA-seq. *Science* **347**, 1138-1142, doi:10.1126/science.aaa1934 (2015).

5 Tsai, H.-H. *et al.* The Chemokine Receptor CXCR2 Controls Positioning of Oligodendrocyte Precursors in Developing Spinal Cord by Arresting Their Migration. *Cell* **110**, 373-383, doi:https://doi.org/10.1016/S0092-8674(02)00838-3 (2002).

6 Wang, D., Sai, J. & Richmond, A. Cell surface heparan sulfate participates in CXCL1-induced signaling. *Biochemistry* **42**, 1071-1077, doi:10.1021/bi026425a (2003).

7 Zhang, Y. *et al.* An RNA-sequencing transcriptome and splicing database of glia, neurons, and vascular cells of the cerebral cortex. *J Neurosci* **34**, 11929-11947, doi:10.1523/JNEUROSCI.1860-14.2014 (2014).
