# Supplementary material for: Overcoming the inhibitory microenvironment surrounding oligodendrocyte progenitor cells following experimental demyelination
Source: Nat Commun. 2021 Mar 26;12:1923. doi: 10.1038/s41467-021-22263-4 (PMC7998003; doi:10.1038/s41467-021-22263-4)
Supplement: Supplementary file 2 — Reporting summary [file 41467_2021_22263_MOESM2_ESM.pdf]

nature research

Corresponding author(s): Sim, Fraser

Last updated by author(s): Jan 25, 2021

Reporting Summary

Nature Research wishes to improve the reproducibility of the work that we publish. This form provides structure for consistency and transparency in reporting. For further information on Nature Research policies, see Authors & Referees and the Editorial Policy Checklist .

Statistics

For all statistical analyses, confirm that the following items are present in the figure legend, table legend, main text, or Methods section.

n/a	Confirmed	
☐	☒	The exact sample size (n) for each experimental group/condition, given as a discrete number and unit of measurement
☐	☒	A statement on whether measurements were taken from distinct samples or whether the same sample was measured repeatedly
☐	☒	The statistical test(s) used AND whether they are one- or two-sided<br>*Only common tests should be described solely by name; describe more complex techniques in the Methods section.*
☐	☒	A description of all covariates tested
☐	☒	A description of any assumptions or corrections, such as tests of normality and adjustment for multiple comparisons
☐	☒	A full description of the statistical parameters including central tendency (e.g. means) or other basic estimates (e.g. regression coefficient) AND variation (e.g. standard deviation) or associated estimates of uncertainty (e.g. confidence intervals)
☐	☒	For null hypothesis testing, the test statistic (e.g. F, t, r) with confidence intervals, effect sizes, degrees of freedom and P value noted<br>*Give P values as exact values whenever suitable.*
☒	☐	For Bayesian analysis, information on the choice of priors and Markov chain Monte Carlo settings
☒	☐	For hierarchical and complex designs, identification of the appropriate level for tests and full reporting of outcomes
☒	☐	Estimates of effect sizes (e.g. Cohen's d, Pearson's r), indicating how they were calculated

Our web collection on statistics for biologists contains articles on many of the points above.

Software and code

Policy information about availability of computer code

Data collection	Micromanager (version 1.4). cellSens (Olympus, version 2.2). Primer Express (v1, Applied Biosystems, CA).
Data analysis	R (4.0.2)/Bioconductor (3.12). GraphPad Prism (San Diego, CA)(9.0.0). FIJI (https://fiji.sc/) (ImageJ 1.53c). Python (3.8). Excel (Office 365).

For manuscripts utilizing custom algorithms or software that are central to the research but not yet described in published literature, software must be made available to editors/reviewers. We strongly encourage code deposition in a community repository (e.g. GitHub). See the Nature Research guidelines for submitting code & software for further information.

Data

Policy information about availability of data

All manuscripts must include a data availability statement. This statement should provide the following information, where applicable:

- Accession codes, unique identifiers, or web links for publicly available datasets
- A list of figures that have associated raw data
- A description of any restrictions on data availability

Source data for all figures and supplemental figures is made available in the source data file. All other supporting data (i.e. images and other data) of the current study are available from the corresponding author upon reasonable request.

Field-specific reporting

Please select the one below that is the best fit for your research. If you are not sure, read the appropriate sections before making your selection.

☒ Life sciences ☐ Behavioural & social sciences ☐ Ecological, evolutionary & environmental sciences

Life sciences study design

All studies must disclose on these points even when the disclosure is negative.

Sample size	Sample size was based on previous experience and published data that utilize the lysolecithin model for assessment of remyelination (Fancy et al., 2009, Mei et al., 2014) and cell culture experiments utilizing human primary glia (e.g. Sim et al., 2011, Wang et al., 2014, Pol et al., 2018, Welliver et al., 2018)
Data exclusions	No data was excluded from these analyses.
Replication	All attempts at replication were successful. Animals from multiple independent litters were used for each in vivo experiment. All experiments involving human cells were replicated on isolates from separate samples (i.e. different individuals) from at least three individual donors.
Randomization	All human tissue samples and cells were used based on availability. Selection of human samples was random. The selection of animals for these studies was randomized. Small molecule administration was randomized.
Blinding	The analysis of demyelinated lesions in Sulf1/2 cKO and PI-88 treated animals was done in a blinded fashion. For human in vitro experiments, sample identity was not blinded as this was not possible. Experiments are replicated in separate culture vessel and in time preventing blinding. For counting of in vitro cell phenotypes (O4/GFAP), conditions were blinded prior to counting. Blinding was not relevant for experiments involving unbiased automated counts (i.e. luciferase or migration assessment). Blinding of human MS vs. control tissue was not possible due to the gross changes in tissue appearance.

Reporting for specific materials, systems and methods

We require information from authors about some types of materials, experimental systems and methods used in many studies. Here, indicate whether each material, system or method listed is relevant to your study. If you are not sure if a list item applies to your research, read the appropriate section before selecting a response.

Materials & experimental systems

n/a	Involved in the study	
☐	☒	Antibodies
☐	☒	Eukaryotic cell lines
☒	☐	Palaeontology
☐	☒	Animals and other organisms
☐	☒	Human research participants
☒	☐	Clinical data

Methods

n/a	Involved in the study	
☒	☐	ChIP-seq
☒	☐	Flow cytometry
☒	☐	MRI-based neuroimaging

Antibodies

Antibodies used

Primary Antibodies:

Rabbit anti-NG2 Chondroitin Sulfate Proteoglycan antibody (Millipore, Cat# AB5320, RRID:AB_11213678, 1:200 dilution)

Rabbit anti-Olig-2 antibody (Millipore, Cat# AB9610, RRID:AB_570666,1:500 dilution).

Mouse anti-GFAP monoclonal antibody (Sigma-Aldrich, Cat# G3893, RRID: AB_477010-1:500 dilution).

Mouse anti-GFAP monoclonal antibody (Biolegend, Covance Research Products Inc, Cat# SMI-21R-100, RRID:AB_509978-1:300 dilution).

Mouse anti-APC (Ab-7) (CC-1) monoclonal antibody (Millipore, Cat# OP80, RRID:AB_2057371, 1:50 dilution).

Rabbit anti-Iba1 antibody (Wako Chemicals USA, Cat# 019-19741, RRID:AB_839504,1:300 dilution).

Rabbit anti-cleaved caspase-3 (Asp175) antibody (Cell Signaling Technology, Cat# 9661, RRID:AB_2070042,1:250 dilution)

HS3A8V (RB4CD12) phage display antibody (gift of Toin van Kuppevelt, Nijmegen Medical Center, Nijmegen, Netherlands, 1:10 dilution).

Mouse O4 IgM hybridoma supernatant (gift of Dr. James Goldman, Columbia University, 1:25 dilution).

Mouse anti-Sulfatase 2 (2B4) monoclonal antibody (AbCam, Cat# ab113405, RRID:AB_10861018, 1:500 dilution).

Rabbit anti-Phospho-Smad1/5 (Ser463/465) (41D10) monoclonal antibody (Cell Signaling Technology, Cat# 9516, RRID:AB_491015, 1:1000 dilution).

Mouse anti-Active-beta-Catenin (anti-ABC) (clone 8E7) monoclonal antibody (Millipore, Cat# 05-665, RRID:AB_309887, 1:2000 dilution).

Mouse anti-Beta-Actin (8H10D10) monoclonal antibody (Cell Signaling Technology, Cat# 3700, RRID:AB_2242334, 1:10,000).

Mouse anti-6X His tag (HIS.H8) monoclonal antibody (AbCam, Cat# ab18184, RRID:AB_444306,1-700 dilution).

Secondary antibodies:

Goat Anti-Rabbit IgG (H+L) Antibody, Alexa Fluor 594 Conjugated (Molecular Probes Cat# A-11012, RRID:AB_141359, 1-500 dilution).

Goat Anti-Mouse IgG (H+L) Highly Cross-adsorbed Antibody, Alexa Fluor 488 Conjugated (Molecular Probes Cat# A-11029, RRID:AB_138404,1-500 dilution).

Goat Anti-Rabbit IgG (H+L) Highly Cross-adsorbed Antibody, Alexa Fluor 647 Conjugate (Molecular Probes Cat# A-21245, RRID:AB_141775, 1-500 dilution),

IRDye 800CW Goat anti-Rabbit IgG antibody (LI-COR Biosciences Cat# 926-32211, RRID:AB_621843,1:5000 dilution),

IRDye 680RD Donkey anti-Mouse IgG antibody (LI-COR Biosciences Cat# 926-68072, RRID:AB_10953628, 1:5000 dilution),

Validation

With the exception of RB4CD12 antibody, all of the antibodies used in the manuscript are all commercially available. All the antibodies used here have been well characterized and validated by the providers or previous publications. Below are the providers' links to the antibody information and relevant publications.

Anti-NG2 antibody. From the manufacturer: AB5320 identifies both the intact proteoglycan and the core protein by Western blot and ELISA. When oligodendrocyte precursor cells (i.e. O-2A progenitor cells) are stained alive, the stain appears as clusters on the cell surface. This antibody does not stain differentiated oligodendrocytes well. (https://www.emdmillipore.com/US/en/product/Anti-NG2-Chondroitin-Sulfate-Proteoglycan-Antibody,MM_NF-AB5320) (Wang et al., 2018. Cell Reports 25: 3435).

Anti-Olig-2 Antibody was shown to stain Olig2 (Oligodendrocyte transcription factor 2) for use in IC, IH, IH(P), IP and WB in human, mice and rat brain by the manufacturer. Recognizes the ~32 kDa Olig-2 protein by Western blot. (https://www.emdmillipore.com/US/en/product/Anti-Olig-2-Antibody,MM_NF-AB9610) (Welliver et al., 2018. J Neurosci 38:6921).

Mouse anti-GFAP antibody was shown to stain astrocytes in pig, rat and human by the manufacturer. From the manufacturer: The antibody reacts specifically with GFAP in immunoblotting assays and labels astrocytes, Bergmann glia cells and chondrocytes of elastic cartilage in immunohistochemical staining. The antibody reacts with glial specific antigen in frozen or alcohol-fixed tissue sections. (https://www.sigmaaldrich.com/catalog/product/sigma/g3893?lang=en&region=US&cm_sp=Insite-_-caSrpResults_srpRecs_srpModel_g3893-_-srpRecs3-1) (M Brenner et. al., Nature genetics, 27(1), 117-120 (2001-1-4)).

Mouse anti-human GFAP antibody (SMI-21R-100) was shown to stain astrocytes in Human, Monkey, Canine by the manufacturer. From the manufacturer: SMI 21 has been tested for immunocytochemical localization of GFAP in astrocytes and Bergmann glia in human, monkey and dog paraffin sections. Astrocytomas from human and dog show a positive reaction with SMI 21. Rat, rabbit and mouse brain sections do not react with SMI 21. Other species have not been examined. No reactivity was observed on liver and kidney paraffin sections. SMI 21 reacts with GFAP and shows no cross reactivity with other intermediate filaments on tissue sections or Western blots of human brain proteins. Rat cytoskeletal proteins do not react with SMI 21. (https://www.biolegend.com/en-us/products/anti-gfap-antibody-11057) (Benraiss A, et al. 2016. Nat Commun. 7:11758.).

Mouse anti-APC (Ab-7) (CC-1), recognizes APC in oligodendrocytes and astrocytes. It is validated for ICC, IF, free-floating Sections, and paraffin and frozen sections in human ,mice and rat brain by the manufacturer. (https://www.emdmillipore.com/US/en/product/Anti-APC-Ab-7-Mouse-mAb-CC-1,EMD_BIO-OP80). (Bhat, R.V., et al. 1996. Glia 17, 169.)

Rabbit anti-Iba1 was shown to stain microglia in mouse and human brain and spinal cord tissues in rabbit by the manufacturer. From the manufacturer: Wako has launched rabbit polyclonal antibodies were raised against a synthetic peptide corresponding to the Iba1 carboxy-terminal sequence, which was conserved among human, rat and mouse Iba1 protein sequences. Rabbit Anti Iba1 antibody is raised a synthetic peptide corresponding to C-terminus of Iba1. Purified by the antigen affinity chromatography from rabbit antisera and prepared in TBS solution. Contains no preservatives and stabilizers. Reactive with human, mouse and rat Iba1. (https://labchem-wako.fujifilm.com/us/product/detail/W01W0101-1974.html). (Ximerakis, M., et al.: Nat. Neurosci., 10, 1696(2019).)

Rabbit anti-cleaved caspase-3 (Asp175). From the manufacturer: Cleaved Caspase-3 (Asp175) Antibody detects endogenous

levels of the large fragment (17/19 kDa) of activated caspase-3 resulting from cleavage adjacent to Asp175. This antibody does not recognize full length caspase-3 or other cleaved caspases. This antibody detects non-specific caspase substrates by western blot. Non-specific labeling may be observed by immunofluorescence in specific sub-types of healthy cells in fixed-frozen tissues (e.g. pancreatic alpha-cells). Nuclear background may be observed in rat and monkey samples. (https://www.cellsignal.com/products/primary-antibodies/cleaved-caspase-3-asp175-antibody/9661). More than 4500 citations.

HS3A8V (RB4CD12) phage display antibody. Validated in Jenniskens et al., 2000. J Neurosci 20:4099.

O4 IgM hybridoma supernatant. O4 is a widely used antibody that reacts with antigens on the surface of developing oligodendrocytes in a stage-specific manner. Characterized by Rashmi Bansal and Steve Pfeifer (Bansal et al., J Neurosci Res 1989, 24:548). Also see - Welliver et al., 2018. J Neurosci 38:6921.

Mouse anti-sulfatase 2 antibody (2B4). From manufacturer: ab113405 recognises an epitope located in the C terminal domain of Sulfatase 2/SULF2. ab113405 shows no cross-reactivity with Sulfatase 1. Sulfatase 2/SULF2 transfected HEK29 cells or non-small-cell Human lung carcinoma tissue (Lemjabbar et al.,2010. Oncogene 29:635). (https://www.abcam.com/sulfatase-2sulf2-antibody-2b4-ab113405.html)

Rabbit anti-phospho-Smad1/5 (Ser463/465) (41D10) Rabbit mAb detects endogenous levels of Smad1 and Smad5 only when dually phosphorylated at Ser463 and Ser465 and is also predicted to detect Smad9 (Smad8) when phosphorylated at Ser465 and Ser467. The antibody does not cross-react with other Smad-related protein in Human, Mouse, Rat by the manufacturer (Ventura et al., 2018). (https://www.cellsignal.com/products/primary-antibodies/phospho-smad1-5-ser463-465-41d10-rabbit-mab/9516)

Mouse anti-Active-β-Catenin (anti-ABC)(8E7) antibody. From the manufacturer: This antibody was used in immunocytochemistry and showed positive staining on the membrane and cytosol. This antibody has also been reported by an independent laboratory to show positive immunostaining for beta-catenin in LiCl-treated 293T cells fixed with methanol (Staal, Frank J. T., 2002). Recognizes active form of β-catenin, dephosphorylated on Ser37 or Thr41. (https://www.emdmillipore.com/US/en/product/Anti-Active-Catenin-Anti-ABC-Antibody-clone-8E7,MM_NF-05-665).

Mouse anti-beta-actin (8H10D10) monoclonal antibody. From the manufacturer: β-Actin (8H10D10) Mouse mAb detects endogenous levels of total β-actin protein. Due to the high sequence identity between the cytoplasmic actin isoforms, β-actin and cytoplasmic γ-actin, this antibody may cross-react with cytoplasmic γ-actin. It does not cross-react with α-skeletal, α-cardiac, α-vascular smooth, or γ-enteric smooth muscle isoforms. More than 1500 citations in database. (https://www.cellsignal.com/products/primary-antibodies/b-actin-8h10d10-mouse-mab/3700.)

Mouse monoclonal 6X His tag® antibody [HIS.H8]. From the manufacturer: was shown to recognize His-tagged recombinant proteins or His-tagged proteins overexpressed in cells and it reacts to recombinant proteins containing the 6X His tag® or 10X His tag® fused to either the amino or carboxy terminus by the manufacturer (Sophie M. Thompson et.al; 2009). (https://www.abcam.com/6x-his-tag-antibody-hish8-ab18184.html)

Secondary antibodies:

Li-Cor IRDye® 800CW and 680 were shown to use in Western blot and In-Cell Western™ assay applications by the manufacturer.

Alexa 488/594/647-labeled goat anti-rabbit IgG, Alexa 488/594/647-labeled goat anti-mice IgG were shown to immunohistochemically stain the corresponding primary antibodies by the manufacturer.

Eukaryotic cell lines

Policy information about cell lines

Cell line source(s)	1. CG-4 cells (obtained from Steve Goldman, Rochester)<br>2. HEK 293T (obtained from Abdel Benraiss, Rochester)
Authentication	CG-4 cells have been validated in the Goldman lab (while Dr. Sim was a postdoc in this lab) and subsequently in the Sim lab by analysis of gene expression, and assessment of the capacity to differentiate as immature O4+ and MBP+ oligodendrocytes following mitogen withdrawal and as type-2 astrocytes (Gfap+A2b5+) following additional of serum.<br><br>HEK 293T cells were not authenticated beyond routinely establishing that these cells are transfected at high efficiency (mCherry) and produce high viral titers when transfected with appropriate lentiviral backbone and packaging plasmids.
Mycoplasma contamination	CG-4 and HEK 293T cell lines are not routinely tested for mycoplasma contamination.
Commonly misidentified lines (See ICLAC register)	No commonly misidentified cell lines were used in this study.

Animals and other organisms

Policy information about studies involving animals; ARRIVE guidelines recommended for reporting animal research

Laboratory animals	This study used mice either C57b6 or BALB/C. Animals of both sex were used in these studies and all animals were at least 8 weeks of age prior to starting the procedures. Animals were typically group housed with appropriate environmental enrichment and maintained in 12 hour light/dark cycle with set points at 73 degrees Fahrenheit and 50 % humidity.
Wild animals	Study did not involve wild animals.
Field-collected samples	Study did not involve field collected samples.
Ethics oversight	University at Buffalo IACUC approved all protocols prior to commencing these studies

Note that full information on the approval of the study protocol must also be provided in the manuscript.

Human research participants

Policy information about studies involving human research participants

Population characteristics	MS TISSUE - The incidence of MS is twice as common in females as males and the overall breakdown of samples collected in the tissue repository is representative of a standard US multiple sclerosis population.<br><br>FETAL TISSUE - No information available. The population characteristics is not available to the investigator.
Recruitment	MS TISSUE - All patients were recruited as part of the MS tissue donation program approved by the Cleveland Clinic Institutional Review Board.<br><br>FETAL TISSUE - Subjects with obvious malformations or previously known genetic disorders are excluded from the study.
Ethics oversight	MS TISSUE - MS tissue was collected as part of the tissue procurement program approved by the Cleveland Clinic Institutional Review Board. All experiments were carried out in accordance with the relevant Cleveland Clinic Institutional regulations and guidelines. The reported work utilized post mortem tissue specimens from expired donors that are stored in this MS tissue bank. Individuals involved in reported study did not have access to areas of the database that contain patient identifiable private information. For all of these reasons, it is our interpretation that the research on MS specimens proposed in this study does not involve human subjects. These specimens do not fulfill the definitions of "human subjects" because there is never any interaction with the living individual patient and "the use of autopsy materials is not directly regulated by 45 CFR Part 46."<br><br>FETAL TISSUE - University at Buffalo, Human Subjects-Institutional Research Board (HS-IRB).<br>The HSIRB reviewed the above referenced project and determined that the research project does not involve human subjects as defined under HHS regulations 45 CFR 46,102 (f). Specimens will not be collected specifically for his proposed research project and we cannot readily ascertain the identity to which the specimens pertain.

Note that full information on the approval of the study protocol must also be provided in the manuscript.
